# A Systematic Review and Meta-Analysis of Practices Exposing Humans to Avian Influenza Viruses, Their Prevalence, and Rationale

**DOI:** 10.4269/ajtmh.17-0014

**Published:** 2017-05-15

**Authors:** Guillaume Fournié, Erling Høg, Tony Barnett, Dirk U. Pfeiffer, Punam Mangtani

**Affiliations:** 1 Veterinary Epidemiology, Economics and Public Health Group, Department of Pathobiology and Population Sciences, Royal Veterinary College, Hatfield, United Kingdom; 2 Department of Global Health and Development, London School of Hygiene and Tropical Medicine, London, United Kingdom; 3 School of Veterinary Medicine, City University of Hong Kong, Kowloon, Hong Kong; 4 Department of Infectious Disease Epidemiology, London School of Hygiene and Tropical Medicine, London, United Kingdom

## Abstract

Almost all human infections by avian influenza viruses (AIVs) are transmitted from poultry. A systematic review was conducted to identify practices associated with human infections, their prevalence, and rationale. Observational studies were identified through database searches. Meta-analysis produced combined odds ratio estimates. The prevalence of practices and rationales for their adoptions were reported. Of the 48,217 records initially identified, 65 articles were included. Direct and indirect exposures to poultry were associated with infection for all investigated viral subtypes and settings. For the most frequently reported practices, association with infection seemed stronger in markets than households, for sick and dead than healthy poultry, and for H7N9 than H5N1. Practices were often described in general terms and their frequency and intensity of contact were not provided. The prevalence of practices was highly variable across studies, and no studies comprehensively explored reasons behind the adoption of practices. Combining epidemiological and targeted anthropological studies would increase the spectrum and detail of practices that could be investigated and should aim to provide insights into the rationale(s) for their existence. A better understanding of these rationales may help to design more realistic and acceptable preventive public health measures and messages.

## Introduction

All four of the influenza virus strains that resulted in pandemics in the last century have had an avian origin. While the 1918–1919 H1N1 pandemic strain was entirely derived from an avian virus,^1^ the subsequent pandemic strains of H2N2 in 1957, H3N2 in 1968, and H1N1 in 2009 all acquired gene segments from avian viruses by reassortment.^2^^,^^3^ Within the last 20 years, a variety of avian influenza virus (AIV) subtypes affecting domestic poultry—especially H5N1, H7N9, and H9N2—has resulted in human infections in mainly Asia and Egypt.^4^^–^^6^ Although these zoonotic transfers are sporadic and their transmission is not sustained within human populations, they also show a potential for reassortment with human viruses^7^; a very few nucleotide substitutions in some circulating strains might allow them to be transmissible between humans.^8^ It is widely feared that ongoing circulation of zoonotic AIVs within poultry populations and their transfer to humans could result in emergence of a novel human pandemic strain. As almost all human cases result from exposure to poultry or to environments contaminated by poultry,^9^^–^^11^ mitigation measures intended to prevent zoonotic infections and reduce the risk of adaptation of these viruses to human hosts must be carefully targeted, not only toward the poultry populations sustaining these viruses^12^ but also toward practices exposing people to infected poultry and contaminated environments. Mitigation measures have to take into account the complexity and difficulty of behavior change strategies and techniques, recognizing that “behavior” should not be construed as exclusively “individual” but as located within a socioeconomic and cultural milieu.

This study presents a systematic review of the scientific literature relating to practices exposing humans to AIVs in Asia and Egypt. The objectives of the review are to identify poultry exposure practices associated with human infection, describe their prevalence within human populations, and examine the rationales for their persistence. This review is informed by insights from social anthropology. It recognizes that the analytical category “practice(s)” as deployed in the literature reviewed largely ignores the social, economic, and cultural context(s) and the subjective meanings of such “practices” for the “practitioners.”

## Materials and Methods

### Search strategy and selection criteria.

This systematic review adheres to PRISMA guidelines (see checklist in Supplemental Material).^13^ A database search of MEDLINE, Science Citation Index, Social Science Citation Index, and The Zoological Record was conducted during the period October 10, 2014, to January 12, 2015. The search used the Boolean search criteria “A AND B”, as follows:

A: “avian influenza” OR “avian flu” OR “bird flu” OR “influenza A” OR “H5N1” OR “H7N7” OR “H7N9” OR “H9N2” and
B: “animal-human” OR “backyard farms” OR “biosecurity” OR “chicken farms” OR “commercial farms” OR “cultural practices” OR “disease transmission” OR “duck farms” OR “exposure” OR “face masks” OR “farms” OR “gloves” OR “human exposure” OR “human infection” OR “live bird markets” OR “live poultry markets” OR “market practices” OR “markets” OR “occupational exposure” OR “poultry farms” OR “prevention” OR “risk” OR “risk + exposure” OR “risk behavior” OR “risk practices” OR “seroconversion” OR “seroprevalence” OR “transmission.”

The “Title”, “Keywords,” and “Abstract” fields were selected in all databases, except for the MEDLINE database which offered to search “All Fields.” EndNote was used to manage citations and remove duplicates.

Eligible articles had to be published between January 1, 1997, and December 31, 2014. This review start date was chosen because the first H5N1 human case was reported during that year.^14^ Articles had to either assess 1) the association between poultry exposure practices and clinical or asymptomatic infection by AIVs (hereafter referred to as risk factor studies) or 2) the prevalence of these practices in human populations (hereafter referred as practice prevalence studies). The poultry exposure practices under consideration had to result in physical contact with poultry or contact with environments potentially contaminated by poultry. Human infections with AIVs could be either clinical cases that were laboratory confirmed or seropositive, asymptomatic individuals. If the association between a practice and human infection was assessed, a measure of effect had to be reported.

Practice prevalence studies included studies assessing proportions of individuals adopting defined practices in a given population, as well as studies only mentioning the presence or absence of defined practices in the study population. For both study types, searches were restricted to English-language publications and studies based in Asia and Egypt, where the subtypes currently causing most human cases (H5N1, H9N2, and H7N9) are endemic.^4^^–^^6^ In addition, we assessed all studies identified in the initial search if they explored the reasons why people adopt practices, which may promote human exposure to avian influenza, whether they gave quantitative information on risk factors for infection or prevalence of practices.

Screening of titles and abstracts was carried out by one reviewer and checked by a second to remove studies unlikely to contain relevant information. Where exclusion could not be justified by one reviewer based solely on screening of a record's title and abstract, the full text was retrieved to allow both reviewers to reach a consensus.

### Data analysis.

The quality of included risk factor studies was assessed using an adaptation of the Cochrane Risk of Bias Tool.^15^ Risk of bias was assessed for the following domains: bias due to confounding, bias in the selection of participants into the study, bias in measurement of exposures, bias due to missing data and bias in measurement of outcome. Based on these domain-level assessments, the overall risk of bias of each study was assessed as low, moderate, serious, or critical (Supplemental Text 1, Supplemental Table 1). The quality of the practice prevalence studies was based on the rigor of the sampling strategy and the representativeness of the findings either at the province or country level. To be classified as Quality 1, participants had to be recruited using random sampling at the provincial (first administrative division) or national level. If sampling was not random (e.g., purposive and convenience) and/or the study was conducted at the level of a district (second administrative division) or lower, the study was classified as Quality 2.

For all studies, the following variables were extracted: study period, location, study design, study population, sample size, setting (household, live bird market [LBM], and farm). For risk factor studies, the following variables were also extracted: case definitions, poultry exposure practices measured, and their associated non-adjusted and adjusted measure of association with outcomes (e.g., odds ratio [OR]) along with their 95% confidence interval (CI) and *P* value. For practices that were investigated in two or more studies, we examined heterogeneity between studies using the *I*^2^ statistic,^16^ and computed overall OR estimates using the random-effect model of DerSimonian and Laird.^17^ As adjusted ORs were not reported in all studies, ORs that were not adjusted for other exposures were used as model inputs. A sensitivity analysis was conducted by differentiating studies according to their risk of bias and locations (Supplemental Text 2, Supplemental Tables 2 and 3).

For the practice prevalence studies, we focused on those practices identified a priori to be associated with human infection. For each practice, the proportion of people or households adopting it was extracted, along with the associated 95% CI. When the CI of a proportion was not mentioned in a paper, the binomial proportion CI (also referred to as the exact method) was calculated. These practices included raising poultry at home, keeping birds inside the house, visiting LBMs, touching poultry during purchase, handling (touching, selling, throwing, and incinerating) or eating sick or dead poultry, slaughtering poultry, and using personal protective equipment (PPE). We did not aim to compute overall estimates for the prevalence of each practice, but rather to describe variations in prevalence estimates for given practices across settings and studies. The range and median of reported prevalences and *I*^2^ statistic were reported. If some practice prevalence studies explored reasons why people adopted some of the practices of interest based on responses to interviews and observations, these rationales were extracted. Data were extracted by a first reviewer and then checked for missing data and inaccuracies by a second reviewer. A sensitivity analysis was conducted by differentiating studies according to their geographical location and their quality score (Supplemental Text 2, Supplemental Tables 4 and 5).

All analyses were run using R 3.2.2^18^ and the package “metafor”.^19^

### Roles of the funding source.

The funders of the study had no role in study design, data collection, data analysis, data interpretation, or writing of the report. The corresponding author had full access to all the data in the study and had final responsibility for the decision to submit for publication.

## Results

### Study selection and characteristics.

**Figure 1.**
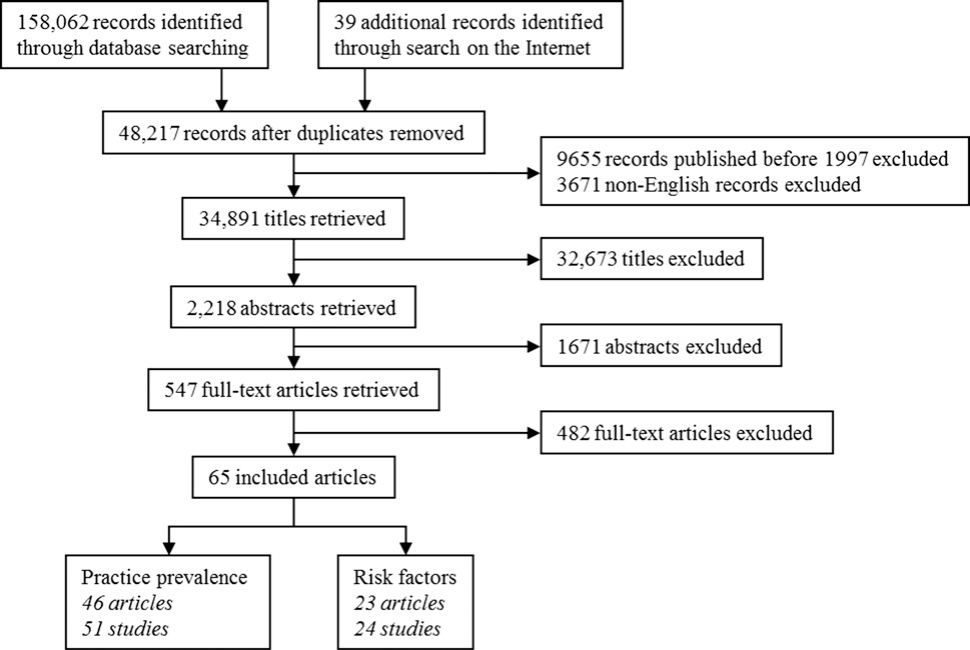
Flow of selected studies.

In total, 547 full texts were screened. Of these, 65 articles were included in the systematic review (Figure 1). Some articles reported multiple studies conducted in different countries over different years, targeting different populations (e.g., households and market workers) or focusing on different virus subtypes, and exploring both the presence of risk factors and the prevalence of practices. They were considered as separate studies. Twenty-three articles incorporated 24 *risk factor studies* (Table 1) and 46 articles presented 51 *practice prevalence studies* (Supplemental Table 8).

**Table 1:** Characteristics of studies exploring risk or protective factors for avian influenza virus infection in humans

Reference	Location	Year(s)	Design	Study population and selection criteria	*N* (cases)	Case definition
						*Incident cases of laboratory confirmed H5N1 clinical infection*
Mounts and others^20^	China (HK)	1997	CC	H5N1 clinical cases, controls matched by age, sex, location	56 (15)	H5N1 clinical infection: respiratory illness and either a viral culture or a 4-fold rise in Ab titer
Zhou and others^21^	China	2005–2008	CC	H5N1 clinical cases, controls matched by age, sex, location	162 (28)	H5N1 clinical infection: respiratory illness and either a viral culture, a 4-fold rise in Ab titer or a RT-PCR
Areechokchai and others^22^	Thailand	2004	CC	H5N1 clinical cases, controls matched by age, location	80 (16)	H5N1 clinical infection: respiratory illness and viral culture or a RT-PCR
Dinh and others^23^	Vietnam	2004	CC	H5N1 clinical cases, controls matched by age, sex, location	134 (28)	H5N1 clinical infection: respiratory illness and a RT-PCR
Yupiana and others^24^	Indonesia	2005–2008	E	H5N1 clinical cases	34 (34)	H5N1 clinical infection: respiratory illness and either a viral culture, a 4-fold rise in Ab titer, a RT-PCR or MN Ab titer ≥ 1:80 and another serological test
						*Prevalent cases of laboratory confirmed H5N1 asymptomatic infection*
Vong and others^25^	Cambodia	2006	CC	H5N1 seropositive rural residents, controls matched by age, sex, location and H5N1 poultry flock status	31 (7)	H5N1 seropositive: MN Ab titer ≥ 1:80 and Western blot
Bridges and others^26^	China (HK)	1997–1998	CC	Poultry workers	1312 (81)	H5N1 seropositive: MN Ab titer ≥ 1:80 and Western blot
Cavailler and others^27^	Cambodia	2007	CS	Rural residents	700 (18)	H5N1 seropositive: MN Ab titer ≥ 1:80 and HI Ab titer ≥ 1:160
Huo and others^28^	China	2010	CS	Residents raising poultry nearby lakes with wildfowl	306 (8)	H5N1 seropositive: HI Ab titer ≥ 1:160
Li and others^29^	China	2010–2012	CS	Poultry workers	1169 (55)	H5N1 seropositive: HI Ab titer ≥ 1:160 and MN
Gomaa and others^30^	Egypt	2010–2011	CS	Rural residents	708 (15)	H5N1 seropositive: MN Ab titer ≥ 1:80 and HI test
						*Incident cases of laboratory confirmed H7N9 clinical infection*
Li and others^31^	China	2013	CC	H7N9 clinical cases, controls matched by age, sex, location	100 (25)	H7N9 clinical infection: respiratory illness and either a viral culture, a 4-fold rise in Ab titer or a RT-PCR
Liu and others^32^	China	2013	CC	H7N9 clinical cases, controls matched by age, sex, location	429 (89)	H7N9 clinical infection: respiratory illness and either a viral culture, a 4-fold rise in Ab titer or a RT-PCR
He and others^33^	China	2013	CC	H7N9 clinical cases, controls matched by age, sex, location	258 (43)	H7N9 clinical infection: respiratory illness and either a viral culture, a 4-fold rise in Ab titer or a RT-PCR
Ai and others^34^	China	2013	CC	H7N9 clinical cases, controls matched by age, sex, location	118 (25)	H7N9 clinical infection: respiratory illness and a RT-PCR
Yu and others^35^	China	2013	E	H7N9 clinical cases	60 (60)	H7N9 clinical infection: respiratory illness and either a viral culture, a 4-fold rise in Ab titer or a RT-PCR. Eighteen rural cases, two cluster cases and four mild cases were excluded.
Fang and others^36^	China	2013	E	H7N9 clinical cases	113 (113)	H7N9 clinical infection: respiratory illness and either a viral culture, a 4-fold rise in Ab titer or a RT-PCR
Fuller (2014)^37^	China	2013	E	Individuals with respiratory illness tested for H7N9	NS	H7N9 clinical infection: respiratory illness and either a viral culture, or a RT-PCR
Wu and others^38^	China	2013–2014	E	H7N9 clinical cases	182 (182)	H7N9 clinical infection: respiratory illness and either a viral culture, a 4-fold rise in Ab titer, or a RT-PCR
						*Incident cases of laboratory confirmed H7N9 asymptomatic infection*
Wang and others^39^	China	2013	C	Poultry market workers in districts with H7N9-infected market	96 (52)	H7N9 seroconversion: HI Ab titer ≥ 1:40 in December, and a ≥ 4-fold rise from May to December.
						*Prevalent cases of laboratory confirmed H7 asymptomatic infection*
Ahad and others^40^	Pakistan	2010–2011	CS	Chicken farm workers	354 (120)	H7 seropositive: HI Ab titer ≥ 1:160
						*Prevalent cases of laboratory confirmed H9 asymptomatic infection*
Yang and others^41^	China	2011	CS	Duck farm workers	1741 (12)	H9 seropositive: HI Ab titer ≥ 1:40
Yu and others^42^	China	2009–2010	CS	Poultry farm and abattoir workers	305 (14)	H9 seropositive: 2 MN Ab titer ≥ 1:80
Gomaa and others^30^	Egypt	2010–2011	CS	Rural residents	648 (38)	H9 seropositive: MN Ab titer ≥ 1:80

Ab titer = antibody titer; C = cohort; CC = case–control; CS = cross-sectional; E = ecological; HI = hemagglutination inhibition; HK = Hong Kong; MN = microneutralization assay; PCR = polymerase chain reaction. In case definition, respiratory illness includes pneumonia and influenza-like illness.

Twenty of the 24 risk factor studies investigated either H5N1 (*N* = 11) or H7N9 (*N* = 9) infections. Three studies detected H9 and one detected H7. Cases were defined as patients with a clinically apparent infection—as opposed to asymptomatic infection—in half of H5N1 studies and in all but one H7N9 studies. Most studies had either a case–control (*N* = 10) or a cross-sectional (*N* = 8) study design. Four out of the five ecological studies focused on H7N9, and there was only one cohort study. Half of the H5N1 studies and all H7N9 studies were conducted in China (including Hong Kong). Other study sites were Cambodia, Egypt, Indonesia, Pakistan, Thailand, and Vietnam. Six studies focused on workers in commercial poultry farms (as opposed to household flocks), markets, and/or abattoirs. The other 18 studies recruited participants from the general or rural populations. The number of cases in the 10 case–control studies ranged from 7 to 89, with a median of 27. In seven of the eight cross-sectional studies, the prevalence of infection was lower than 6%. The quality assessment of these studies is detailed in Supplemental text 1. Ten studies were assessed as being at moderate risk of bias and 14 at serious risk of bias.

All 51 practice prevalence studies were cross-sectional and conducted in 14 countries, mostly in southeast Asia (*N* = 25, 49%) and China (*N* = 11, 22%, including Hong Kong). Thirty-six studies explored poultry exposure practices in households, whereas practices adopted by poultry market, farm, and/or abattoir workers were explored in 15 studies. All but three studies explored practices using standardized questionnaires. The remaining three studies, conducted in Bangladesh, used observations and in-depth interviews. Sample sizes were highly variable, ranging from 34 to 4950, with a median of 312. We classified 21 studies as Quality 1 and 30 studies as Quality 2 (Supplemental Table 8).

### Association between poultry exposure practices and AIV infection.

**Figure 2.**
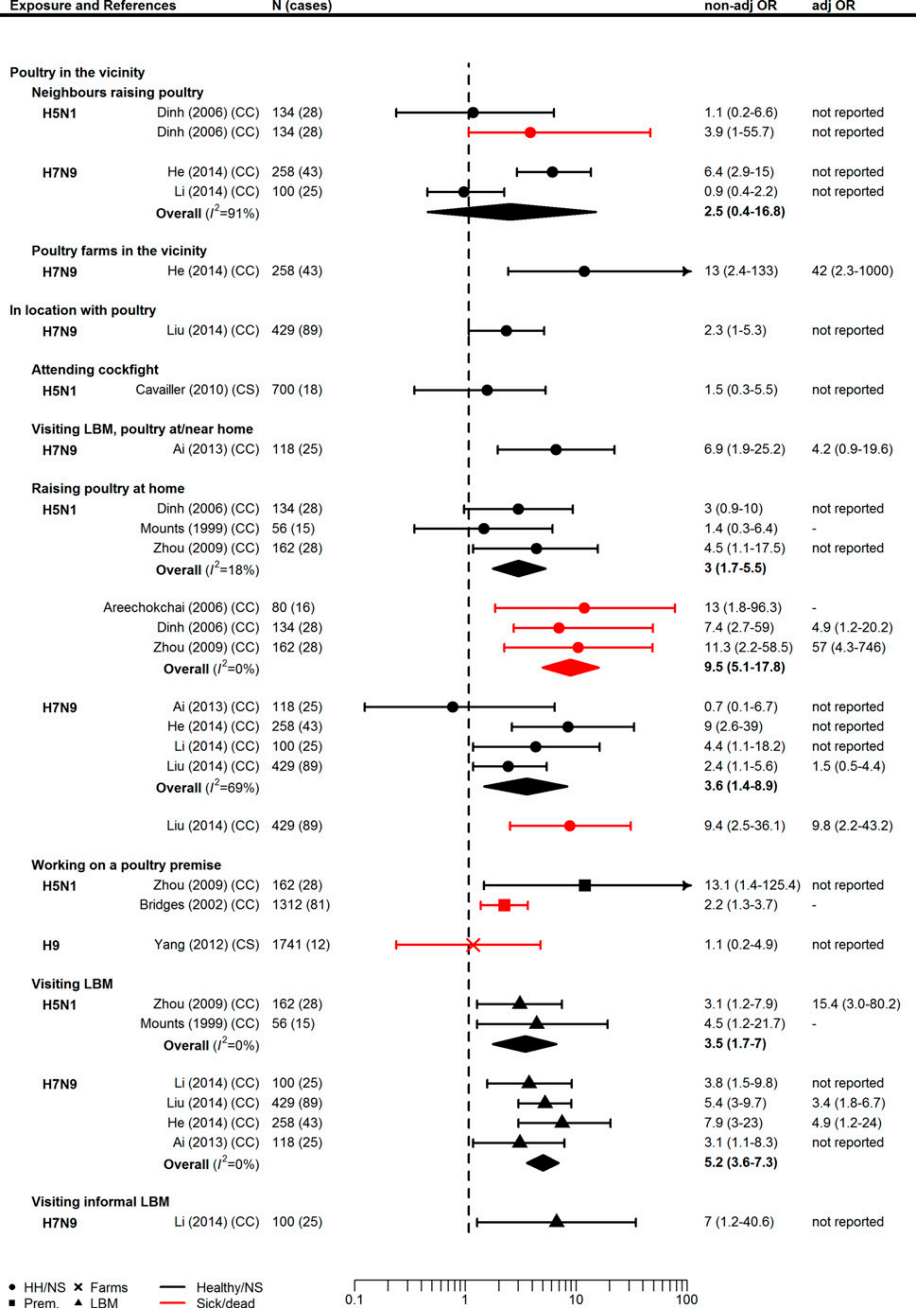
Association between human AIV infection and indirect exposure to poultry in various locations. HH = Household; NS = not specified; Prem = premises, include farms, markets and abattoirs; non-adj OR= odds ratio is not adjusted for other exposure practices; adj OR = odds ratio is adjusted for other exposure practices; not reported = multivariate analysis was conducted but adjusted OR was not reported for the practice of interest. This figure appears in color at www.ajtmh.org.

**Figure 3.**
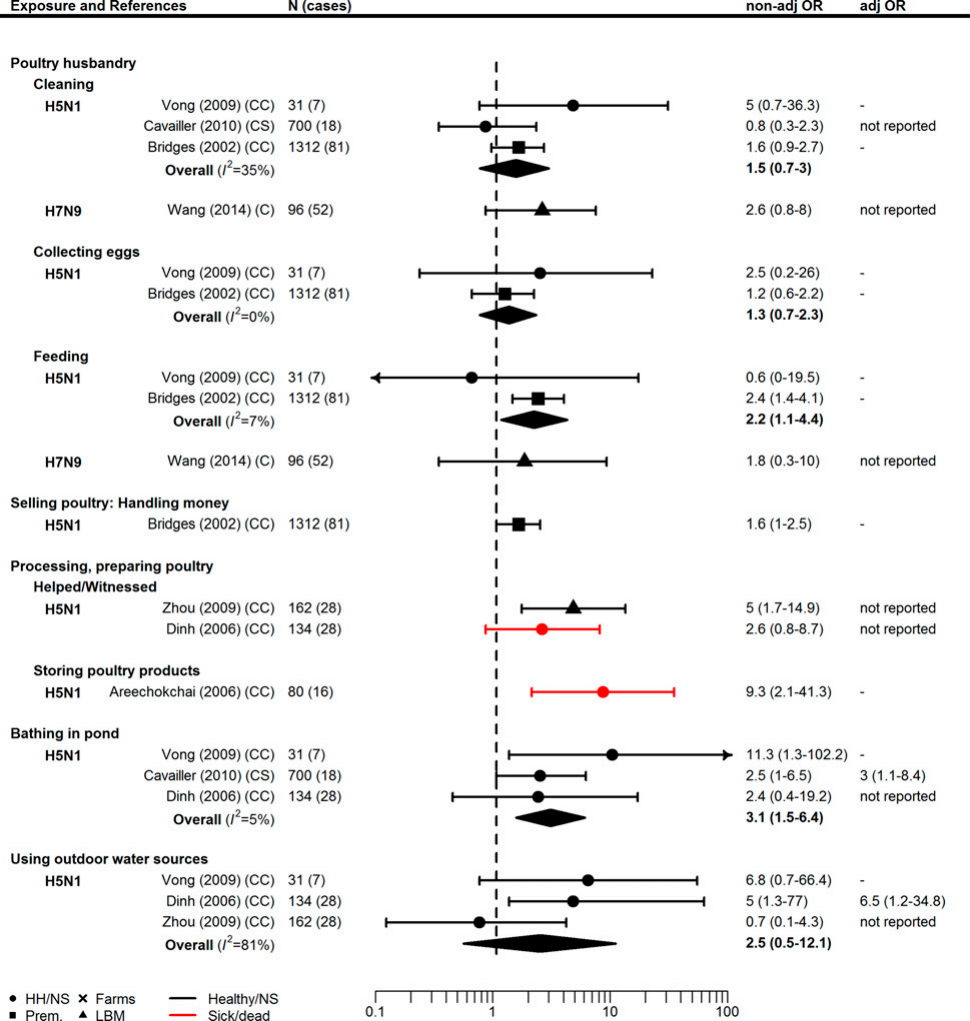
Association between human AIV infection and practices resulting in indirect exposure. HH = household; NS = not specified; Prem = premises, include farms, markets and abattoirs; non-adj OR = odds ratio is not adjusted for other exposure practices; adj OR = odds ratio is adjusted for other exposure practices; not reported = multivariate analysis was conducted but adjusted OR was not reported for the practice of interest. This figure appears in color at www.ajtmh.org.

**Figure 4.**
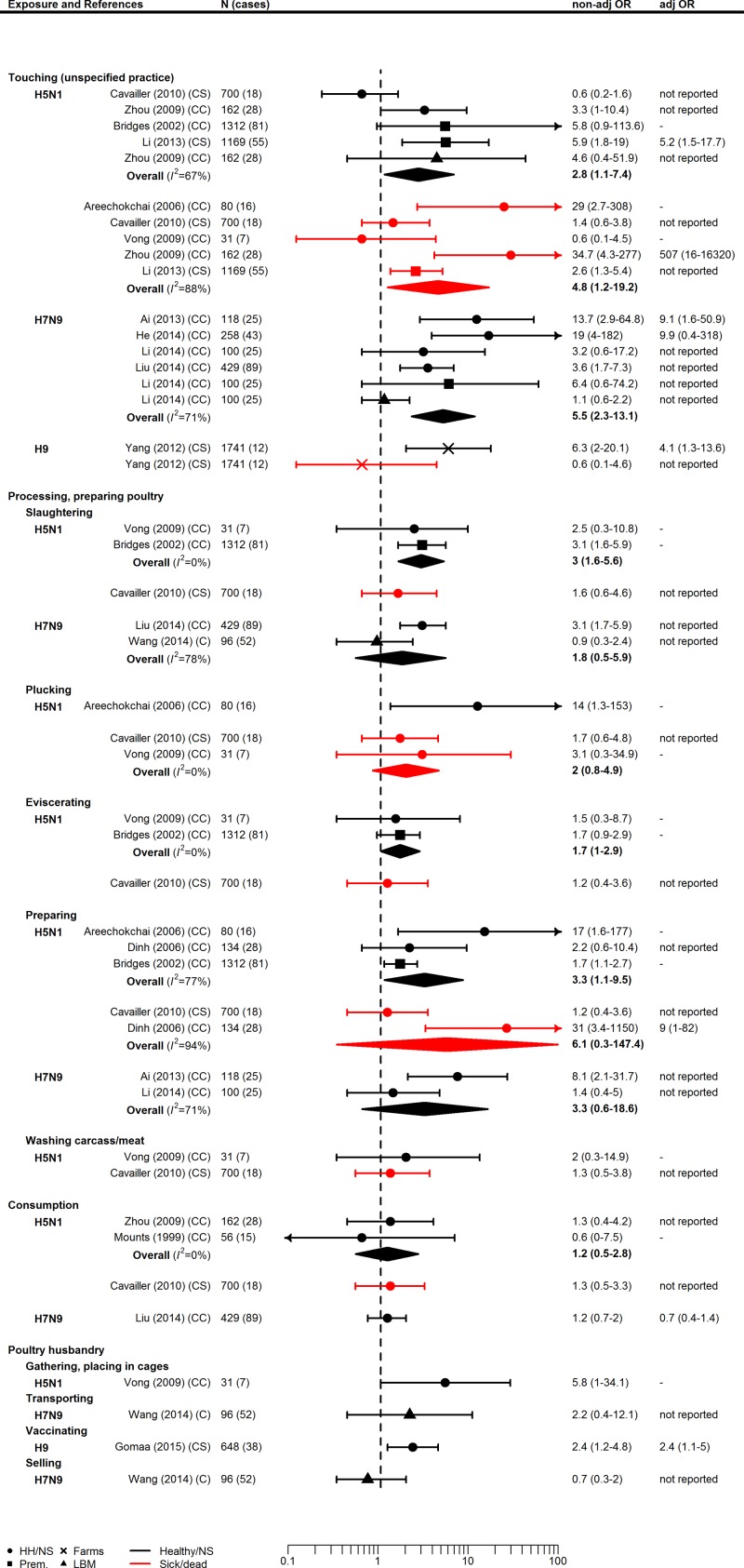
Association between human AIV infection and practices resulting in direct exposure. HH = household; NS = not specified; Prem = premises, include farms, markets and abattoirs; non-adj OR = odds ratio is not adjusted for other exposure practices; adj OR = odds ratio is adjusted for other exposure practices; not reported = multivariate analysis was conducted but adjusted OR was not reported for the practice of interest. This figure appears in color at www.ajtmh.org.

**Figure 5.**
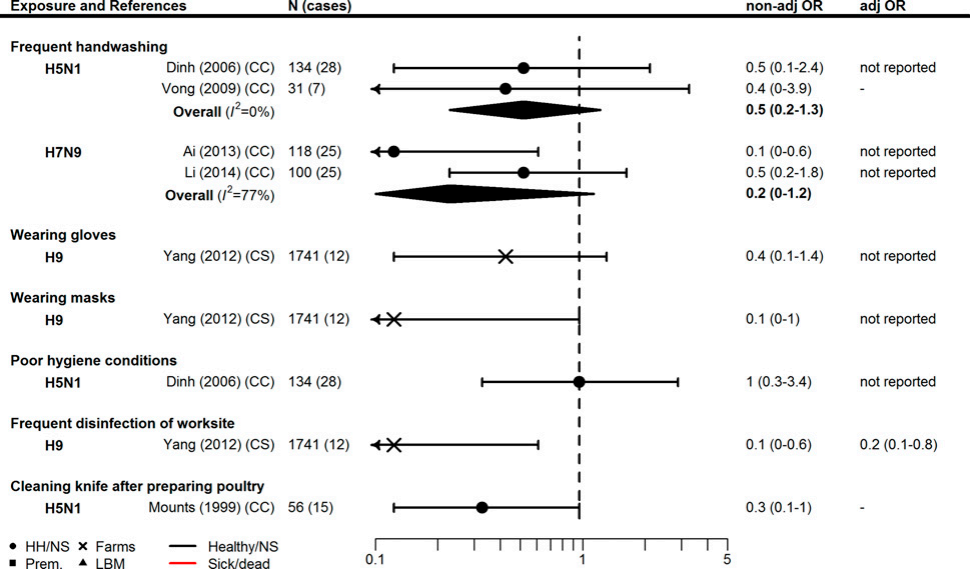
Association between human AIV infection and preventive practices. HH = household; NS = not specified; Prem = premises, include farms, markets and abattoirs; non-adj OR = odds ratio is not adjusted for other exposure practices; adj OR = odds ratio is adjusted for other exposure practices; not reported = multivariate analysis was conducted but adjusted OR was not reported for the practice of interest. This figure appears in color at www.ajtmh.org.

Study-specific and pooled ORs for each poultry exposure practice explored in the included case–control, cohort, and cross-sectional studies are shown in Figures 2–5 (detailed exposures in Supplemental Table 12).

#### Indirect exposure.

Indirect exposure to poultry was generally expressed as the co-occurrence of poultry and study participants in a given environment: within the neighborhood, at home, in a LBM, or at the worksite. Poultry could be described as healthy, sick, or dead. The evidence for an association between infection and presence of poultry in backyard or commercial farms in the vicinity of study participants' homes was variable across studies (Figure 2). Meta-analysis results suggested that the presence of poultry at home substantially increased the odds of infection by H5N1 (pooled OR = 3, 95% CI = 1.7–5.5) and H7N9 (pooled OR = 3.6, 95% CI = 1.4–8.9). Heterogeneity was, however, large among H7N9 studies (*I*^2^ = 69%). Odds of infection were even further increased if poultry raised at home became sick or died (H5N1, pooled OR = 9.5, 95% CI = 5.1–17.8).

Occupational exposure to poultry was explored in only three studies, of which two suggested an association with human infection.^21^^,^^26^ Visits to LBMs were associated with infection in all studies reporting such exposure. The pooled OR estimate for H7N9 infection was 5.2 (95% CI = 3.6–7.3) and for H5N1 3.5 (95% CI = 1.7–7). These results were in agreement with ecological study findings (Supplemental Table 6). H5N1 infection in Indonesia was associated with the occurrence of poultry outbreaks in the same area (RR = 1.3, 95% CI = 1.1–17).^24^ Two studies found an association between H7N9 cases and the density of LBMs,^36^^,^^37^ whereas two others noted a drastic reduction in H7N9 incidence with LBM closure.^35^^,^^38^

Increase in the odds of infection with the proximity between poultry and humans, the size of the susceptible poultry population, and the frequency of exposure was further suggested through the exploration of additional variables (Supplemental Table 7). Keeping poultry cages inside rather than outside the home^21^ and raising larger backyard^28^ and commercial^29^ flocks with suboptimal vaccination coverage^21^ increased the odds of infection.

In three studies, ORs were shown to increase with the frequency of visits to LBMs.^21^^,^^31^^,^^34^ The effect of occupational exposure was found to further vary with premise type: breeder and layer farms were at higher risk for their employees than other farm types in two studies,^40^^,^^41^ whereas working in retail markets was riskier than in wholesale markets and farms in another study.^26^

Regarding poultry species, there was weak evidence in one study that the OR of H5N1 infection was higher when raising waterfowl at home than when raising chickens only.^21^ In one study, exposure to geese and turkeys, respectively, increased the odds of H5N1 infection, whereas exposure to ducks increased the odds of H9 infection.^30^ However, the odds of H9 infection were not different as between duck and chicken keepers in another study^42^ (Supplemental Table 7).

Only seven out of the 19 case–control, cohort, and cross-sectional studies described specific activities leading to indirect exposures. Most studies found weak evidence of association between infection and poultry husbandry-related activities (Figure 3). Feeding poultry in farms, witnessing poultry slaughter in markets, and storing products from sick/dead poultry at home were found to be associated with H5N1 infection, each of them in one study. Some investigated activities were unrelated to poultry management but to contact with water potentially contaminated by poultry. Although pooled OR estimates revealed weak evidence of an association between H5N1 infection and the use of outdoor water (pooled OR = 2.5, 95% CI = 0.5–12.1), the evidence was stronger for bathing in water bodies (pooled OR = 3.1, 95% CI = 1.5–6.4).

#### Direct exposure.

As with indirect exposure, direct exposure to poultry was often described in general terms as direct contact with, touching or handling poultry. Meta-analysis suggested an association between handling poultry and infection (Figure 4). Heterogeneity was high among studies and could not only be explained by the diversity of settings, as the level of heterogeneity was not reduced when only considering households (Supplemental Table 3). This association seemed slightly stronger for H7N9 (pooled OR = 5.5, 95% CI = 2.3–13.1) than H5N1 (pooled OR = 2.8, 95% CI = 1.1–7.4), and when sick or dead poultry were concerned (H5N1, pooled OR = 4.8, 95% CI = 1.2–19.2). However, handling poultry did not seem to result in higher odds of infection than indirect exposure at home or in markets.

The investigated activities resulting in direct exposure to poultry were related to poultry husbandry, processing, and consumption (Figure 4). While consumption of healthy-appearing or sick poultry was not associated with H5N1 or H7N9 infection, several stages of poultry processing—such as slaughtering, evisceration, and preparation—were found in the meta-analysis to increase the odds of H5N1 infection. Regarding poultry husbandry practices, vaccinating and handling birds to place them into cages were found associated with H9 and H5N1 infection in two different studies. Although a study found weak evidence for higher odds of H9 infection among chicken butchers than keepers (OR = 3.4, 95% CI: 0.8–14.5),^42^ the small number of studies for each processing and husbandry activity, and the often high level of heterogeneity among them limit the comparisons that can be performed across activities, viral subtypes and poultry health status.

#### Protective factors.

Regarding hygiene practices (Figure 5), meta-analysis provided weak evidence for frequent handwashing being a protective factor (H5N1 pooled OR = 0.5, 95% CI = 0.2–1.3; H7N9 pooled OR = 0.2, 95% CI = 0–1.2). Only one study examined use of masks and frequent disinfection of worksites (e.g., farms). It noted a reduced odds of infection (ORs = 0.1, 95% CI = 0–1 and ORs = 0.1, 95% CI = 0–0.6, respectively).^41^

### Prevalence of poultry exposure practices.

Exposure to live birds was widespread in studied populations (detailed prevalences in Supplemental Table 9). Although the proportion of households raising backyard poultry was higher in rural than in peri-urban and urban areas, purchasing live poultry in markets was more frequent in peri-urban and urban rather than rural settings (Table 2, Supplemental Figures 1 and 2). Levels of contact with poultry at markets and households greatly varied across studies: the proportion of households keeping poultry inside their own house ranged from 1% to 87% (Table 2), and the proportion of households slaughtering birds at home ranged from 12% to 85% (Table 3, Supplemental Figure 3). The proportion of respondents who reported touching poultry when purchasing it in markets was lower than 18% in the three surveys conducted in Hong Kong, whereas it was higher than 58% in five of six surveys conducted in mainland China, Viet Nam, and Thailand (Supplemental Figure 2).

**Table 2:** Prevalence of practices related to backyard poultry rearing and purchase of poultry in LBMs

	All households			Urban households			Peri-urban households			Rural households		
	*n* (c)	p (range)	*I*^2^	*n* (c)	p (range)	*I*^2^	*n* (c)	p (range)	*I*^2^	*n* (c)	p (range)	*I*^2^
Raise backyard poultry	24 (10)	65 (19–96)	99	4 (3)	45 (19–51)	98	3 (2)	50 (34–55)	91	15 (9)	78 (50–96)	98
Keep poultry inside house	6 (4)	20 (1–87)	100									
Visit LBMs to purchase poultry	11 (4)	38 (7–81)	100	3 (1)	38 (33–81)	100	1 (1)	77	–	3 (1)	8 (7–9)	0
Touch when purchasing	9 (4)	59 (5–92)	100									

LBM = live bird market; *n* (c) = number of studies along with the number of countries in which these studies took place in brackets; p (range) = median prevalence (%) and range.

**Table 3:** Prevalence of preventive practices and practices related to the slaughtering and processing of poultry, and the management of sick and dead poultry

	Households			Premises			Farms			Live bird markets		
	*n* (c)	p (range)	*I*^2^	*n* (c)	p (range)	*I*^2^	*n* (c)	p (range)	*I*^2^	*n* (c)	p (range)	*I*^2^
Slaughtering and processing poultry	11 (7)	45 (12–85)	99				2 (2)	22 (6–38)	98	4 (4)	73 (39–100)	98
Management of sick and dead poultry												
Touching	9 (3)	33 (14–75)	99	2 (1)	11 (8–13)	86				1 (1)	41	–
Consumption	13 (6)	12 (2–100)	99							1 (1)	16	–
Selling	7 (6)	26 (0–100)	99				1 (1)	81	–	2 (2)	28 (2–53)	99
Returning to suppliers				1 (1)	50	–				1 (1)	9	–
Throwing in open spaces	11 (7)	45 (2–87)	99	1 (1)	20	–						
Burying/Incinerating	13 (8)	72 (2–95)	99				2 (2)	62 (30–95)	99			
Preventive practices												
Washing hands with soap after contacts with poultry	6 (4)	97 (4–99)	96							1 (1)	68	–
Wearing gloves	3 (3)	2 (1–2)	0	2 (1)	51 (47–54)	81	4 (4)	18 (1–30)	90	1 (1)	60	–
Wearing facemask	4 (3)	2 (0–2)	0	2 (1)	44 (42–46)	37	4 (4)	21 (6–66)	98	4 (4)	18 (13–45)	93
Wearing aprons/changing clothes	2 (2)	3 (0–5)	0	1 (1)	89	–	3 (3)	4 (3–34)	94	4 (2)	42 (15–80)	96
Wearing boots	1 (1)	6	–				3 (3)	15 (7–16)	6	4 (2)	60 (30–80)	92
Rinsing/washing equipment after use	2 (2)	66 (33–99)	98				1 (1)	41	–	4 (1)	50 (19–100)	97

*n* (c) = number of studies along with the number of countries in which these studies took place in brackets; p (range) = median prevalence (%) and range. Premises include farms, markets, and abattoirs.

The surveyed populations were highly heterogeneous in terms of their management of sick and dead poultry (Table 3). In Bangladesh, Egypt and, to a lesser extent, Cambodia, the proportion of survey participants burying or incinerating dead poultry was generally lower and the proportion consuming, selling, or throwing sick or dead poultry into open spaces was generally higher when compared with other study sites (including China, India, Indonesia, Lao, Thailand, and Viet Nam) (Supplemental Figure 4).

The proportion of household survey participants reporting handwashing with soap after contact with poultry was higher than 80% in all studies exploring this practice, except in one study in Bangladesh where the proportion dropped to 4%. The adoption of PPE was higher among farm and market workers than in households. However, most workers generally reported not wearing PPE (Supplemental Figure 5).

Some additional practices were reported by only a couple of studies, including practices exposing humans to potentially contaminated environments. This included cleaning places where poultry are kept, bathing in water bodies in Cambodia, washing carcasses in water bodies in Cambodia and Bangladesh, and barefoot contact with blood in Bangladesh.

### Rationale for poultry exposure practices.

None of the reviewed studies sought to explore the rationales behind practices at risk of human exposure to AIVs. Eight practice prevalence studies did address some of the reasons for conducting some practices, but not comprehensively (Supplemental Table 10). Other studies discussed these rationales as post hoc hypotheses (Supplemental Table 11). However, exploring rationales requires targeted research and in none of the papers were these dealt with according to the canons of social anthropology and ethnography.

The eight prevalence studies, in which reasons for some practices were briefly discussed, took place in Bangladesh, Egypt, Indonesia, Lao, and Turkey. In Bangladesh, backyard farmers reported keeping poultry in their bedroom because they are concerned about predation and thieves.^43^ Some kept sick poultry in their bedroom to separate them from their healthy poultry and to keep them under observation.^44^ This practice was related to the perception that people are not at risk of illness. Some people may have been aware of avian influenza, but they considered the risks associated with poultry handling as negligible. Moreover, several practices were described by the authors as a matter of preference, rooted in tradition (based on interviews of residents or developed as post hoc hypotheses).^45^ For instance, live poultry bought in markets were preferred to chilled or frozen meat for consumption as the latter was considered to be of lower quality.^46^ Poultry slaughtered immediately before cooking were traditionally believed to be fresher, more flavorsome and nutritious, and less likely to be contaminated.^47^ Likewise, reasons for touching poultry before buying were related to consumer traditions, relying on their own judgment of the quality and safety of the poultry.^47^ Authors also related the adoption of certain risky practices to poverty. High poultry prices meant that purchasing poultry meat was not affordable for the poorest, and thereby encouraged the consumption of sick or dead poultry.^44^^,^^47^^,^^48^

The non-adoption of preventive measures was mainly explained by authors by financial constraints, such as implementation costs and potential impact on business, absence of supporting legislation, time and space constraints, and “risk fatigue” from repeated outbreaks.^45^^,^^49^^–^^52^ This is generally analyzed in the context of poor populations that do not consider avian influenza to be a major health threat, and for whom the perceived chance of an adverse outcome from poultry exposure is considered to be relatively low compared with the adverse outcome of worsening poverty.

Heterogeneity in practices across settings was often explained by authors by differing religious beliefs. In Bangladesh, sick poultry were slaughtered and consumed if it was thought that they were about to die because of religious bans on eating animals that die of natural causes.^44^^,^^48^ In contrast, consumption of dead poultry was reported in non-Muslim populations. According to Buddhist principles, killing is considered to have karmic consequences. Thai people were therefore considered to be less likely to slaughter poultry themselves.^47^

## Discussion

Both direct and indirect exposures to poultry in households, farms, or markets were associated with human infection by AIVs in most of the reviewed risk factor studies. The strength of this association seemed stronger for H7N9 than for H5N1, for sick and dead compared with healthy poultry, and in markets compared with any other setting. Several studies also suggested that the odds of infection further increased with the proximity between humans and poultry, the size of the poultry population to which humans were exposed, and the frequency of exposure. Direct exposure was not associated with higher odds of infection than indirect exposure. This apparent association between AIV infection and indirect exposure to poultry, and the possible role of handwashing and environmental disinfection as protective factors suggest that contacts with contaminated environments followed by ingestion, intranasal or conjunctival self-inoculation of the virus may be a major mode of AIV transmission. Infected poultry shed a high viral load, which may survive in the environment for a few days under favorable conditions.^53^ In households, virus survival in the environment may represent an infection pressure to which people may have prolonged contact, in particular when poultry are kept inside home, including in bedrooms. Even when environmental exposure is of a shorter duration, such as in the case of people visiting markets, the frequent introduction of infected poultry in markets and the associated viral circulation among marketed poultry^54^ means that humans may be exposed to high virus loads. However, high uncertainty remains regarding the actual modes of transmission involved. Contributions of aerosols and large droplets cannot be ruled out, as investigated exposures may be associated with several modes of transmission.^55^ H5N1 was shown to be transmitted between poultry by aerosols.^56^ Some practices, such as mechanical defeathering, may generate contaminated aerosols and large droplets, and result in the infection of people visiting markets.^57^ These results suggest that interventions aiming to reduce virus load in markets,^58^ and behavioral change strategies leading to higher biosafety standards when handling poultry, especially sick or dead specimens, could substantially reduce human exposure to AIVs. The adoption of risky and protective practices varied greatly across studies, and was frequently explained as motivated by financial constraints and religious beliefs. These variations could also result from temporal changes in people's perception of their risk of infection. As these factors were expected to vary across the heterogeneous socioeconomic and cultural landscape covered by the reviewed articles, risk mitigation interventions should be tailored to these local contexts. However, none of the studies reviewed here aimed to assess the rationale behind practices at risk of human exposure to AIVs. Reviewed knowledge, attitude, and practice studies investigated questions related to awareness and knowledge, and a few studies did touch on some reasons behind specific practices and discussed these as post hoc hypotheses, but neither in sufficient detail nor at the appropriate level of conceptualization.

## Limitations

Our review was exposed to recall bias, as exposures were captured in all risk factor studies, and most practice prevalence studies, through structured interviews of study participants, or proxies when study participants have died. Bias in the measurement of exposures was more pronounced in risk factor studies using serology to define prevalent cases: AIVs being endemic in most settings, there was uncertainty about whether the reported exposures preceded, or not, the infection. However, this bias might be limited as most investigated exposures were daily, routine practices, which might not greatly change in the medium-term in AIV-endemic settings.

The use of structured interviews to measure exposure in all risk factor studies and most practice prevalence studies implied further limitations. Although the observed heterogeneity in the prevalence of practices was generally explained, as mentioned earlier, by variations in socioeconomic and cultural landscapes, this pattern could also result from the limited representativeness induced by the geographically small study sites, and response bias. Asking about past behavior or about practices that may be officially banned or enforced by regulation, or of which their adoption may be positively or negatively perceived by people may in addition result in biased answers, leading to an underestimation of the real levels of exposure. For instance, high compliance to handwashing was reported in all questionnaire surveys that investigated this practice, but it was only actually done by a small proportion of participants observed in one study.^48^ Moreover, structured questionnaire surveys can only investigate the adoption of practices of which the study designers have an awareness. For instance, several practice prevalence studies explored whether participants wear boots dedicated to the care of poultry. Based on observations of poultry rearing by Bangladeshi rural communities, one study was able to identify that people stepped barefoot into poultry blood.^43^ Stepping barefoot in poultry blood might be a more relevant practice than the failure to wear special boots dedicated to poultry care.

The pooled OR estimates often relied on a small number of heterogeneous studies, and, therefore, need to be interpreted with caution. Also, terms used to refer to practices were often only merely named or briefly described. The same term could have different uses, and therefore, different meanings across studies. Multiple specific practices may have been encompassed within these descriptions. Only a few specific practices were investigated, but the frequency and intensity of contact were not detailed, preventing further discrimination of practices according to the degree of exposure to poultry. Overall, investigated practices—or their descriptions in the literature which are not the same as the practices themselves—appear not to have changed significantly within the last 17 years. Investigations have yet to be comprehensive, in-depth analyses of given and related practices. For example, while visiting live bird markets was found to be a risk factor for AIV infection in the first risk factor study in 1997, subsequent studies were rarely able to explore in more detail which types of practices within markets could lead to infection, given the retrospective nature of outbreak investigations.

## Future Research

To address these limitations, epidemiological surveys could benefit from being combined with anthropological investigations. Anthropological studies may help to identify practices that would be better described with observations and in-depth interviews to develop a more accurate and detailed understanding. Such practices may, for instance, include handwashing and the use of PPEs. Moreover, the development of structured interview protocols would greatly benefit from a prior anthropological exploration of both the conceptualization of “practices” and of practices of interest. Whether “practices” are of interest may alter in the light of more detailed description and contextualization. Practices that are not systematically investigated, but which may reveal to be of epidemiological importance, may thus be characterized. Further description of practices could include a characterization of the contacts involved, and a measure of their intensity and frequency. The more detailed and grounded into the local contexts these descriptions are, the less comparable they may be across settings characterized by heterogeneous populations. On the other hand, the more general these descriptions are, the less likely it would be possible to tailor interventions to the local contexts that shape those practices. Detailed descriptions are required to identify the most relevant practices and populations at risk that should be targeted by risk mitigation strategies. Nevertheless, the small number of cases identified in most risk factor studies may limit the exploration of the association between AIV infection and specific, detailed practices. If these practices were only adopted by a small fraction of the population, the statistical power would be low, and even if the actual association with AIV infection was strong, the measured strength of association would be uncertain.

Epidemiological studies typically investigate the most significant causal relationships between practices and the human exposure to AIVs as part of outbreak investigations and interventions to control them. Although causal relationships are difficult to identify, they nevertheless seek to achieve this by examining associations between an exposure variable and a health outcome.^59^^,^^60^ However, identifying the specific practices promoting AIV transmission to humans is often not enough to improve preventive public health interventions and messages. For these messages to be heard and strategies to be adhered to by populations at risk, their design needs to be informed by a thorough understanding of the factors and theories influencing persistence of risky or preventive practices. Of use in such investigations is the rationale behind certain risky practices for exposure to disease: Why do people do what they do? Under which circumstances do they engage in risky practices? At what point do practices become habitual behavior as opposed to a conscious decision in light of the level of risk of pathogen transmission? How do these practices relate to the tradition, culture, and socioeconomic circumstances? How are these practices influenced by disease awareness and knowledge? These questions were not part of the main objectives of reviewed studies. Contextual research appeared to be merely a by-product. As mentioned earlier, practices and their rationales were often only briefly described. These “descriptions” may on closer examination, and informed by ethnographic studies, turn out to have been dealing in homogenizing “labels” rather than in “heterogenizing” “descriptions.” This is important because terms such as “fresh,” “nutritious,” “quality,” and “wholesome”, are each supported by an implicit local “theory” of these things, their importance and significance. Understanding these theories would help to tailor interventions to local circumstances (which may vary within countries), increasing their acceptance in populations at risk. A challenge to achieve this is to access the relation between individual actions and the socioeconomic and cultural environment in which those actions are situated and produced. Recent theoretical^61^^,^^62^ and empirical works explored the use of emergent properties, such as “hope” or “disgust,” as quantitative variables capturing people's experiences of the social, economic, and cultural world they inhabit. In Uganda, the level of hope that a person experienced was measured and found to be associated with some known risk factors for HIV infection.^63^ In India, disgust was associated with handwashing behavior.^64^ Applying this approach may provide new insights about practice adoption and inform the development of preventive public health messages. Such messages would aim to alter the level of a given emergent property (e.g., “hope” and “disgust”) in the targeted population to promote the uptake of protective practices.

In conclusion, the descriptions of practices exposing humans to poultry and their shared environment are often general and with little information to aid understanding of underlying rationales, limiting their usefulness for developing effective control and preventive measures. The assessment of the prevalence of reported practices in populations at risk was also prone to biases acting to underestimate the actual level of exposure. Epidemiological surveys aiming to explore potentially infectious contacts at the human–animal interface would greatly benefit from being combined with anthropological investigations. Such an approach would not only allow a more accurate identification and detailed description of risky as well as preventive practices, but would also allow the exploration of the reasons behind these practices. This would in turn facilitate the development of preventive public health measures and messages more likely to lead to positive behavior change in targeted populations.

## Supplementary Material

Supplemental Figures.

## References

[1] Webster RG Bean WJ Gorman OT Chambers TM Kawaoka Y, 1992 Evolution and ecology of influenza A viruses. Microbiol Rev 56: 152–179. 1579108 10.1128/mr.56.1.152-179.1992 PMC372859

[2] Kawaoka Y Krauss S Webster RG, 1989 Avian-to-human transmission of the PB1 gene of influenza A viruses in the 1957 and 1968 pandemics. J Virol 63: 4603–4608. 2795713 10.1128/jvi.63.11.4603-4608.1989 PMC251093

[3] Smith GJ, 2009 Origins and evolutionary genomics of the 2009 swine-origin H1N1 influenza A epidemic. Nature 459: 1122–1125. 19516283 10.1038/nature08182

[4] Freidl GS, 2014 Influenza at the animal-human interface: a review of the literature for virological evidence of human infection with swine or avian influenza viruses other than A(H5N1). Euro Surveill 19: 20793. 24832117 10.2807/1560-7917.es2014.19.18.20793

[5] Khan SU Anderson BD Heil GL Liang S Gray GC, 2015 A systematic review and meta-analysis of the seroprevalence of influenza A(H9N2) infection among humans. J Infect Dis 212: 562–569. 25712969 10.1093/infdis/jiv109 PMC4598807

[6] World Health Organization (WHO), 2015 Cumulative Number of Confirmed Human Cases for Avian Influenza A(H5N1) Reported to WHO, 2003–2015. Available at: http://www.who.int/influenza/human_animal_interface/EN_GIP_20150717cumulativeNumberH5N1cases.pdf?ua=1. Accessed August 13, 2015.

[7] Li C Hatta M Nidom CA Muramoto Y Watanabe S Neumann G Kawaoka Y, 2010 Reassortment between avian H5N1 and human H3N2 influenza viruses creates hybrid viruses with substantial virulence. Proc Natl Acad Sci USA 107: 4687–4692. 20176961 10.1073/pnas.0912807107 PMC2842136

[8] Russell CA, 2012 The potential for respiratory droplet-transmissible A/H5N1 influenza virus to evolve in a mammalian host. Science 336: 1541–1547. 22723414 10.1126/science.1222526 PMC3426314

[9] Rabinowitz P Perdue M Mumford E, 2010 Contact variables for exposure to avian influenza H5N1 virus at the human-animal interface. Zoonoses Public Health 57: 227–238. 19486500 10.1111/j.1863-2378.2008.01223.x

[10] Van Kerkhove MD Mumford E Mounts AW Bresee J Ly S Bridges CB Otte J, 2011 Highly pathogenic avian influenza (H5N1): pathways of exposure at the animal-human interface, a systematic review. PLoS One 6: e14582. 21283678 10.1371/journal.pone.0014582 PMC3025925

[11] Lai S, 2016 Global epidemiology of avian influenza A H5N1 virus infection in humans, 1997–2015: a systematic review of individual case data. Lancet Infect Dis 16: e108–e118. 27211899 10.1016/S1473-3099(16)00153-5 PMC4933299

[12] Costard S Fournie G Pfeiffer DU, 2014 Using risk assessment as part of a systems approach to the control and prevention of HPAIV H5N1. EcoHealth 11: 36–43. 24488190 10.1007/s10393-014-0907-1

[13] Moher D Liberati A Tetzlaff J Altman DG Group P, 2009 Preferred reporting items for systematic reviews and meta-analyses: the PRISMA statement. PLoS Med 6: e1000097. 19621072 10.1371/journal.pmed.1000097 PMC2707599

[14] Claas EC Osterhaus AD van Beek R De Jong JC Rimmelzwaan GF Senne DA Krauss S Shortridge KF Webster RG, 1998 Human influenza A H5N1 virus related to a highly pathogenic avian influenza virus. Lancet 351: 472–477. 9482438 10.1016/S0140-6736(97)11212-0

[15] Sterne JAC Higgins JPT Reeves BC, 2014 A Cochrane Risk Of Bias Assessment Tool: for Non-Randomized Studies of Interventions (ACROBAT NRSI), Version 1.0.0. *24 September 2014* Available at: sites.google.com/site/riskofbiastool/. Accessed February 1, 2016.

[16] Higgins JP Thompson SG Deeks JJ Altman DG, 2003 Measuring inconsistency in meta-analyses. BMJ 327: 557–560. 12958120 10.1136/bmj.327.7414.557 PMC192859

[17] DerSimonian R Laird N, 1986 Meta-analysis in clinical trials. Control Clin Trials 7: 177–188. 3802833 10.1016/0197-2456(86)90046-2

[18] R Development Core Team, 2015 R: A Language and Environment for Statistical Computing. Vienna, Austria: R Foundation for Statistical Computing.

[19] Viechtbauer W, 2010 Conducting meta-analyses in R with the metafor package. J Stat Software 36: 1–48.

[20] Mounts AW, 1999 Case-control study of risk factors for avian influenza A (H5N1) disease, Hong Kong, 1997. J Infect Dis 180: 505–508. 10395870 10.1086/314903

[21] Zhou L, 2009 Risk factors for human illness with avian influenza A (H5N1) virus infection in China. J Infect Dis 199: 1726–1734. 19416076 10.1086/599206 PMC2759027

[22] Areechokchai D Jiraphongsa C Laosiritaworn Y Hanshaoworakul W, O'Reilly M; Centers for Disease Control and Prevention, 2006 Investigation of avian influenza (H5N1) outbreak in humans–Thailand, 2004. MMWR Morb Mortal Wkly Rep 55: 3–6. 16645574

[23] Dinh PN Long HT Tien NT Hien NT Mai le TQ Phong le H Tuan le V Van Tan H Nguyen NB Van Tu P Phuong NT, 2006 Risk factors for human infection with avian influenza A H5N1, Vietnam, 2004. Emerg Infect Dis 12: 1841–1847. 17326934 10.3201/eid1212.060829 PMC3291373

[24] Yupiana Y de Vlas SJ Adnan NM Richardus JH, 2010 Risk factors of poultry outbreaks and human cases of H5N1 avian influenza virus infection in West Java Province, Indonesia. Int J Infect Dis 14: e800–e805. 20637674 10.1016/j.ijid.2010.03.014

[25] Vong S Ly S Van Kerkhove MD Achenbach J Holl D Buchy P Sorn S Seng H Uyeki TM Sok T Katz JM, 2009 Risk factors associated with subclinical human infection with avian influenza A (H5N1) virus–Cambodia, 2006. J Infect Dis 199: 1744–1752. 19416078 10.1086/599208

[26] Bridges CB, 2002 Risk of influenza A (H5N1) infection among poultry workers, Hong Kong, 1997–1998. J Infect Dis 185: 1005–1010. 11930308 10.1086/340044

[27] Cavailler P Chu S Ly S Garcia JM Ha do Q Bergeri I Som L Sok T Vong S Buchy P, 2010 Seroprevalence of anti-H5 antibody in rural Cambodia, 2007. J Clin Virol 48: 123–126. 20356781 10.1016/j.jcv.2010.02.021

[28] Huo X Zu RQ Qi X Qin YF Li L Tang FY Hu ZB Zhu FC, 2012 Seroprevalence of avian influenza A (H5N1) virus among poultry workers in Jiangsu Province, China: an observational study. BMC Infect Dis 12: 93. 22512873 10.1186/1471-2334-12-93 PMC3348011

[29] Li LH Yu Z Chen WS Liu SL Lu Y Zhang YJ Chen EF Lin JF, 2013 Evidence for H5 avian influenza infection in Zhejiang province, China, 2010–2012: a cross-sectional study. J Thorac Dis 5: 790–796. 24409357 10.3978/j.issn.2072-1439.2013.12.45 PMC3886696

[30] Gomaa MR, 2015 Avian influenza A(H5N1) and A(H9N2) seroprevalence and risk factors for infection among Egyptians: a prospective, controlled seroepidemiological study. J Infect Dis 211: 1399–1407. 25355942 10.1093/infdis/jiu529 PMC4462653

[31] Li J Chen J Yang G Zheng YX Mao SH Zhu WP Yu XL Gao Y Pan QC Yuan ZA, 2015 Case-control study of risk factors for human infection with avian influenza A (H7N9) virus in Shanghai, China, 2013. Epidemiol Infect 143: 1826–1832. 25471822 10.1017/S0950268814003264 PMC9507269

[32] Liu B, 2014 Risk Factors for Influenza A(H7N9) Disease-China, 2013. Clin Infect Dis 59: 787–794. 24928293 10.1093/cid/ciu423

[33] He F Zhang M Wang XY Wu HC Shang XP Li FD Wu C Lin JF Zhu BP, 2014 Distinct risk profiles for human infections with the influenza A(H7N9) virus among rural and urban residents: Zhejiang Province, China, 2013. PLoS One 9: e95015. 24789082 10.1371/journal.pone.0095015 PMC4008429

[34] Ai J, 2013 Case-control study of risk factors for human infection with influenza A(H7N9) virus in Jiangsu Province, China, 2013. Euro Surveillance: Bulletin Europeen sur les Maladies Transmissibles 18: 20510. 10.2807/1560-7917.es2013.18.26.20510 23827526

[35] Yu H, 2014 Effect of closure of live poultry markets on poultry-to-person transmission of avian influenza A H7N9 virus: an ecological study. Lancet 383: 541–548. 24183056 10.1016/S0140-6736(13)61904-2 PMC3946250

[36] Fang LQ Li XL Liu K Li YJ Yao HW Liang S Yang Y Feng ZJ Gray GC Cao WC, 2013 Mapping spread and risk of avian influenza A (H7N9) in China. Sci Rep 3: 2722. 24072008 10.1038/srep02722 PMC3784030

[37] Fuller T Havers F Xu C Fang LQ Cao WC Shu Y Widdowson MA Smith TB, 2014 Identifying areas with a high risk of human infection with the avian influenza A (H7N9) virus in east Asia. J Infect 69: 174–181. 24642206 10.1016/j.jinf.2014.03.006 PMC4077931

[38] Wu P, 2014 Poultry market closures and human infection with influenza A(H7N9) virus, China, 2013–14. Emerg Infect Dis 20: 1891–1894. 25340354 10.3201/eid2011.140556 PMC4214308

[39] Wang X, 2014 Seroprevalence to avian influenza A(H7N9) virus among poultry workers and the general population in southern China: a longitudinal study. Clin Infect Dis 59: e76–e83. 24867786 10.1093/cid/ciu399 PMC4155446

[40] Ahad A, 2014 Risk factors for H7 and H9 infection in commercial poultry farm workers in provinces within Pakistan. Prev Vet Med 117: 610–614. 25457514 10.1016/j.prevetmed.2014.10.007

[41] Yang P, 2012 A serological survey of antibodies to H5, H7 and H9 avian influenza viruses amongst the duck-related workers in Beijing, China. PLoS One 7: e50770. 23226380 10.1371/journal.pone.0050770 PMC3511333

[42] Yu Q, 2013 Risk perceptions for avian influenza virus infection among poultry workers, China. Emerg Infect Dis 19: 313–316. 23343592 10.3201/eid1902.120251 PMC3563274

[43] Sultana R Nahar N Rimi NA Azad S Islam MS Gurley ES Luby SP, 2012 Backyard poultry raising in Bangladesh: a valued resource for the villagers and a setting for zoonotic transmission of avian influenza. A qualitative study. Rural Remote Health 12: 1927. 22950607

[44] Sultana R Rimi NA Azad S Islam MS Khan MSU Gurley ES Nahar N Luby SP, 2012 Bangladeshi backyard poultry raisers' perceptions and practices related to zoonotic transmission of avian influenza. J Infect Dev Ctries 6: 156–165. 22337845 10.3855/jidc.2242

[45] Edirne T Avci DK Dagkara B Aslan M, 2011 Knowledge and anticipated attitudes of the community about bird flu outbreak in Turkey, 2007–2008: a survey-based descriptive study. Int J Public Health 56: 163–168. 20217176 10.1007/s00038-010-0131-x

[46] Ma X, 2014 Knowledge, attitudes and practices relating to influenza A(H7N9) risk among live poultry traders in Guangzhou City, China. BMC Infect Dis 14: 554. 25324011 10.1186/s12879-014-0554-8 PMC4210513

[47] Liao QY Lam WWT Bich TH Dang VT Fielding R, 2014 Comparison of behaviors regarding live poultry exposure among rural residents in Vietnam and Thailand. J Infect Dev Ctries 8: 526–534. 24727520 10.3855/jidc.3545

[48] Rimi NA Sultana R Ishtiak-Ahmed K Khan SU Sharker MAY Uz Zaman R Azziz-Baumgartner E Gurley ES Nahar N Luby SP, 2014 Poultry slaughtering practices in rural communities of Bangladesh and risk of avian influenza transmission: a qualitative study. EcoHealth 11: 83–93. 24306550 10.1007/s10393-013-0885-8 PMC11753187

[49] Negro-Calduch E Elfadaly S Tibbo M Ankers P Bailey E, 2013 Assessment of biosecurity practices of small-scale broiler producers in central Egypt. Prev Vet Med 110: 253–262. 23218658 10.1016/j.prevetmed.2012.11.014

[50] Barennes H Harimanana AN Lorvongseng S Ongkhammy S Chu C, 2010 Paradoxical risk perception and behaviours related to Avian Flu outbreak and education campaign, Laos. BMC Infect Dis 10: 294. 20937155 10.1186/1471-2334-10-294 PMC2959065

[51] Barennes H Martinez-Aussel B Vongphrachanh P Strobe M, 2007 Avian influenza risk perceptions, Laos. Emerg Infect Dis 13: 1126–1128. 18214204 10.3201/eid1307.061197 PMC2878220

[52] Sutanto YC, 2013 Highly Pathogenic Avian Influenza Knowledge, Attitudes, and Practices Study among Live Bird Market Workers in Jakarta—Indonesia. Fort Collins, CO: Department of Clinical Sciences, Colorado State University, 153.

[53] Shortridge KF, 1998 Characterization of avian H5N1 influenza viruses from poultry in Hong Kong. Virology 252: 331–342. 9878612 10.1006/viro.1998.9488

[54] Fournié G Guitian J Mangtani P Ghani AC, 2011 Impact of the implementation of rest days in live bird markets on the dynamics of H5N1 highly pathogenic avian influenza. J R Soc Interface 8: 1079–1089. 21131332 10.1098/rsif.2010.0510 PMC3119874

[55] Tellier R, 2006 Review of aerosol transmission of influenza A virus. Emerg Infect Dis 12: 1657–1662. 17283614 10.3201/eid1211.060426 PMC3372341

[56] Webster RG, 2002 Characterization of H5N1 influenza viruses that continue to circulate in geese in southeastern China. J Virol 76: 118–126. 11739677 10.1128/JVI.76.1.118-126.2002 PMC135698

[57] Allen VM Hinton MH Tinker DB Gibson C Mead GC Wathes CM, 2003 Microbial cross-contamination by airborne dispersion and contagion during defeathering of poultry. Br Poult Sci 44: 567–576. 14584847 10.1080/00071660310001616183

[58] Offeddu V Cowling BJ Malik Peiris JS, 2016 Interventions in live poultry markets for the control of avian influenza: a systematic review. One Health 2: 55–64. 27213177 10.1016/j.onehlt.2016.03.002 PMC4871622

[59] Hill AB, 1965 The environment and disease: association or causation? Proc R Soc Med 58: 295–300. 14283879 10.1177/003591576505800503 PMC1898525

[60] Lucas RM McMichael AJ, 2005 Association or causation: evaluating links between “environment and disease”. Bull World Health Organ 83: 792–795. 16283057 PMC2626424

[61] Aunger R Curtis V, 2015 Gaining Control: How Human Behavior Evolved. Oxford, United Kingdom: Oxford University Press.

[62] Barnett T Fournie G Gupta S Seeley J, 2015 Some considerations concerning the challenge of incorporating social variables into epidemiological models of infectious disease transmission. Glob Public Health 10: 438–448. 25648796 10.1080/17441692.2015.1007155

[63] Barnett T Seeley J Levin J Katongole J, 2015 Hope: a new approach to understanding structural factors in HIV acquisition. Glob Public Health 10: 417–437. 25648679 10.1080/17441692.2015.1007154

[64] Biran A Schmidt WP Varadharajan KS Rajaraman D Kumar R Greenland K Gopalan B Aunger R Curtis V, 2014 Effect of a behaviour-change intervention on handwashing with soap in India (SuperAmma): a cluster-randomised trial. Lancet Glob Health 2: e145–e154. 25102847 10.1016/S2214-109X(13)70160-8

