# Supplementary material for: A Systematic Review and Meta-Analysis of Practices Exposing Humans to Avian Influenza Viruses, Their Prevalence, and Rationale
Source: Am J Trop Med Hyg. 2017 May 15;97(2):376–88. doi: 10.4269/ajtmh.17-0014 (PMC5544094; doi:10.4269/ajtmh.17-0014)
Supplement: Supplementary file 1 [file tpmd170014.SD1.pdf]

SUPPLEMENTARY MATERIAL - A SYSTEMATIC REVIEW AND META-ANALYSIS OF PRACTICES EXPOSING HUMANS TO AVIAN INFLUENZA VIRUSES, THEIR PREVALENCE AND RATIONALE

Authors: Guillaume Fournié, Erling Høg, Tony Barnett, Dirk U Pfeiffer, Punam Mangtani

SUPPLEMENTARY TEXT 1. QUALITY ASSESSMENT OF RISK FACTOR STUDIES.

The quality of included risk factor studies was assessed by adapting the Cochrane Risk of Bias Tool [1]. We evaluated bias in the following domains: bias due to confounding, bias in the selection of participants into the study, bias in measurement of exposures, bias due to missing data, bias in measurement of outcome. For each domain, the following signalling questions were used to inform the judgements:

Bias due to confounding:
- If present, were confounding factors adjusted for?

Bias in the selection of participants into the study:
- (Cohort studies) Were exposed and unexposed participants drawn from the same population?
- (Case-control studies) Were controls sampled from the same population as cases?
- (cross-sectional studies) Were inclusion and exclusion criteria applied uniformly?

Bias in measurement of exposures:
- Are exposures well defined?
- Was information on exposures unaffected by knowledge of the infection status or risk of infection?

Bias due to missing data:
- Were infection and exposure data reasonably complete?

Bias in measurement of outcome:
- (cohort and cross-sectional studies) Was the case definition objective?
- (cohort studies) Can we be confident that the outcome of interest was not present at start of the study?
- (case-control studies) Were the case and control definitions objective and consistent?

Based on these questions, a judgement was made on the extent to which the results of the studies were at risk of bias. The options for the assessment of each domain, and for the overall assessment were: low, moderate, serious and critical risk of bias. If assessment in any domain was serious (or critical) the overall assessment was deemed serious (or critical) otherwise it was deemed moderate unless all domains were assessed as low. The results of the assessment are provided in Supplementary table 1.

Bias due to confounding. All 10 case-control studies either matched controls and cases or stratified the analysis according to participants' age, sex and/or location. However, only 6 case-control studies [2-7] conducted multivariate analysis and presented odds ratios adjusted for other poultry exposures. They were classified as being at moderate risk of bias. Six [8-13] out of the 18 case-control, cross-sectional and cohort studies only provided, for each exposure variable, crude odds ratios which were not adjusted for other exposure variables hence considered as having scope for serious bias from confounding. Because of their ecological nature, the 5 ecological studies were likely to be impacted by biases due to confounding. Ecological studies exploring the impact of live bird market closure on the risk of infection [14, 15] did not assess the influence of some seasonal factors, such as seasonal variations in poultry production and marketing, which were likely to impact on the extent of viral circulation in poultry.

Bias in the selection of participants into the study. All study participants were generally all sampled from the same populations. In two case-control studies patients with respiratory illness were more likely to be tested for H5N1 infection if they reported recent contact with any or sick poultry, which might have biased the results towards those exposures [3, 11]. Convenience sampling was used to recruit participants in one cross-sectional study [16], and was likely to generate a selection bias limiting the external validity of the study. Information about the recruitment of study participants was limited for 3 other studies [17-19]. In a cross-sectional study, a third of recruited participants were excluded from the sample as their questionnaires were judged invalid. If there was an association between exclusion of participants and some features associated with poultry exposures (e.g. level of education, time available to answer questionnaire), this may generate bias limiting the validity of the study [13].

Bias in measurement of exposures. All cohort, case-control and cross-sectional studies assessed exposures using self-report data, therefore, some misclassification of exposures was likely. Moreover, all case-control studies using clinical infection as part of the case definition included a substantial proportion of cases that had died for which information about exposure was obtained through the interview of proxies [2, 4-8, 11, 20]. These proxies might have been less aware of specific activities, resulting in a bias toward the underestimation of the association between an exposure and infection. For studies conducted during new epidemics (e.g. [4, 5, 8, 11]), media reports and high levels of alarm among the public might have led to difference in recall between cases and controls. The substantial delay between measurements of outcome and exposures in most case-control studies was also a source of recall bias [2, 4-8, 10, 20]. In one study, controls were interviewed later than cases, when the public was more aware of the role of poultry exposure as a risk factor for infection. Controls might have been more likely to report such exposures, resulting in an underestimation of the association between exposure and infection [6]. For all studies using serology to define cases, the presence of antibodies may not imply recent exposure given that the virus of interest was endemic in most investigated settings. Therefore, there was uncertainty about whether the reported exposures preceded, or not, the infection. Exposures were assessed in ecological studies using aggregated data, such as density of poultry and density of live bird markets. If the quality of this data was spatially variable, it might

have led to bias. The studies exploring the impact of live bird market closure on the risk of infection [14, 15] assumed that the infection pressure was constant before, and after live bird market closure, respectively. Yet, this infection pressure depended on the prevalence of infection among incoming poultry, which was likely to vary over time [21]. It might have resulted in a miss-estimation of the reduction in the probability of infection.

Bias due to missing data. All studies were judged at low risk of bias due to missing data. Infection and exposure data were reasonably complete, and there was no indication that possible missing data were non-randomly distributed among study participants.

Bias in measurement of outcome. In the cohort study, some participants were seropositive at the start of the follow-up period, but were kept in the cohort [17]. They were therefore classified as having not sero-converted, which might have biased the results towards an underestimation of the association between some exposures and the outcome. In studies using clinical infection as part of the case definition, it was unclear whether other potential disease aetiology which could have affected the case in combination with AIVs (eg Dengue [11]) were investigated, and could have led to a misclassification of these cases. Studies using serological results as a marker of infection could have misclassified cases due to cross-reactivity between viral subtypes. This is more likely to be the case using hemagglutinin inhibition (HI) titres compared to microneutralisation (MN) titres. A serological survey defined individuals as infected based on a low antibody titre threshold, which might have led to false positive [18]. Conversely HI titres are less sensitive than MN titres leading to false negatives. Therefore, the use of HI titres only was considered at moderate risk of bias. While individuals had to be seronegative to be recruited as controls in most case-control studies, the serological status of controls was not assessed in two studies [6, 11]. Spatial and temporal variation in surveillance effectiveness would have impacted the validity of the ecological study results.

Supplemental Table 1

Assessment of study quality

Author (year) and study design	Confounding	Selection of participants	Measurement of exposures	Missing data	Measurement of outcomes	Overall
Mounts (1999) (CC)[8]	Serious	Low	Moderate	Low	Low	Serious
Zhou (2009) (CC)[2]	Moderate	Low	Moderate	Low	Low	Moderate
Areechokchai (2006) (CC)[11]	Serious	Moderate	Moderate	Low	Moderate	Serious
Dinh (2006) (CC)[3]	Moderate	Moderate	Moderate	Low	Low	Moderate
Yupiana (2010) (E)[22]	Serious	Low	Serious	Low	Serious	Serious
Vong (2009) (CC)[10]	Serious	Low	Serious	Low	Moderate	Serious
Bridges (2002) (CC)[9]	Serious	Low	Moderate	Low	Moderate	Serious
Cavailler (2010) (CS)[23]	Moderate	Low	Moderate	Low	Moderate	Moderate
Huo (2012) (CS)[12]	Serious	Low	Moderate	Low	Moderate	Serious
Li (2013) (CS)[24]	Moderate	Low	Moderate	Low	Moderate	Moderate
Gomaa (2015) (CS)[16]	Moderate	Serious	Moderate	Low	Moderate	Serious
Li (2014) (CC)[4]	Moderate	Low	Moderate	Low	Low	Moderate
Liu (2014) (CC)[5]	Moderate	Low	Moderate	Low	Low	Moderate
He (2014) (CC)[6]	Moderate	Low	Moderate	Low	Moderate	Moderate
Ai (2013) (CC)[7]	Moderate	Low	Moderate	Low	Low	Moderate
Yu (2014) (E)[14]	Serious	Low	Serious	Low	Serious	Serious
Fang (2013) (E)[25]	Serious	Low	Serious	Low	Serious	Serious
Fuller (2014) (E)[26]	Serious	Low	Serious	Low	Serious	Serious
Wu (2014) (E)[15]	Serious	Low	Serious	Low	Serious	Serious
Wang (2014) (C)[17]	Moderate	Moderate	Moderate	Low	Moderate	Moderate
Ahad (2014) (CS)[19]	Moderate	Moderate	Moderate	Low	Moderate	Moderate
Yang (2012) (CS)[18]	Moderate	Moderate	Moderate	Low	Serious	Serious
Yu (2013) (CS)[13]	Serious	Moderate	Moderate	Low	Moderate	Serious

CC = case–control; CS = cross-sectional; E = ecological. Low, moderate, and serious refer to the level of risk of bias.

SUPPLEMENTARY TEXT 2. SENSITIVITY ANALYSIS.

For practices which were informed by at least 3 studies, pooled ratio estimates were re-assessed with studies grouped according to their risk of bias, moderate or serious. For 5 out of 6 practices, pooled odds ratios estimated based only on studies at moderate risk of bias were slightly lower than combined estimates (Supplementary table 2). This new estimates did not affect the conclusions we made in the main text. Therefore, we presented combined estimates. In the main text, the estimations of pooled odds ratios were for practices leading to direct exposure to poultry (touching, cleaning, preparing poultry) irrespective of the location of exposure. We re-computed these estimates by stratifying studies according to the location of the exposures of interest, households or premises (i.e. commercial farms, live bird markets and abattoirs). Pooled odds ratio estimates based on study conducted in households were generally higher than the combined estimates (Supplementary table 3). However, their associated confidence intervals were much wider, as the I^2 index also increased, meaning that study-specific odds ratios for households were highly heterogeneous. The small number of studies and the high level of heterogeneity between studies prevent us from drawing general conclusions about a possible increase in the strength of the association between direct exposure and infection when considering direct exposures taking place in households only. Moreover, several studies were assumed to be "household" studies but in fact did not specify the location where participants were exposed to poultry, and might have included some participants which were directly exposed to poultry in farms or markets. For instance, in [5], 6% of cases and 1% of controls were defined as poultry workers (defined as deriving at least half of their income from work involving poultry), and the location where some exposures took place, such as "Poultry contact during slaughtering or processing" and "Poultry consumption" was not specified. Also, some studies only included poultry workers as participants [9, 13, 17-19, 24]. For these studies, all non-exposed participants (i.e. no reported direct exposure with poultry) in the baseline group were at least indirectly exposed to poultry. In contrast, when participants were selected from the general population, some non-exposed participants in the baseline group may not have had any indirect exposure to poultry. This may explain that some pooled odds ratio estimates based on study conducted in premises were lower than the combined estimates (Supplementary table 3).

The range of prevalence of each reported practice was re-assessed based on the countries where the studies took place (Supplementary table 4). For some practices, it appeared that most of the heterogeneity across studies could be partially explained by their geographical location. For instance, the proportion of study participants reporting burying/incinerating dead poultry ranged from 2% to 95%. However, the prevalence estimated in the 3 studies conducted in Bangladesh ranged between 2%-12%, and between 16%-19% in the 2 studies conducted in Egypt. It was much higher in Lao and Vietnam, ranging between 78%-87% and 76%-79%, respectively. However, it has to be kept in mind that some research groups produced several estimates for a given practice within a given country. Any differences in prevalence may therefore be more likely to be real as while these estimates were produced at different times, the study population may have remained the same, and the study protocol may be unchanged. As discussed in the main manuscript, the way in which prevalence were assessed–generally based on self-report–was likely to affect the accuracy of any estimates. Finally, the ranges of prevalence did not differ between the 2 study quality groups for most practices (Supplementary table 5). The proportion of participants who reported "selling sick and dead birds" and "throwing dead and sick birds in open spaces" was lower for quality 1 than quality 2 studies. It has to be noted that this quality assessment was only based on whether sampling was random and if regionally or nationally representative or not. Based on the information provided in the articles, it was not possible to assess the reliability of the answers provided by respondents.

Supplemental Table 2

Sensitivity analysis of risk factor studies according to their overall risk of bias

Practice	Subtype	Health status	Sub-group	*n*	pOR (95% CI)	I^2
Raising poultry at home	H5N1	Healthy/NS	All studies	3	3 (1.7–5.5)	18
			Moderate bias	2	3.7 (2–6.7)	0
			Serious bias	1	1.4 (0.3–6.4)	-
Raising poultry at home	H5N1	Sick/dead	All studies	3	9.5 (5.1–17.8)	0
			Moderate bias	2	8.4 (4–17.7)	0
			Serious bias	1	13 (1.8–96.3)	-
Raising poultry at home	H7N9	Healthy/NS	All studies	4	3.6 (1.4–8.9)	69
			Moderate bias	4	3.6 (1.4–8.9)	69
			Serious bias	0	–	–
Visiting LBM	H7N9	Healthy/NS	All studies	4	5.2 (3.6–7.3)	0
			Moderate bias	4	5.2 (3.6–7.3)	0
			Serious bias	0	–	–
Poultry husbandry: Cleaning	H5N1	Healthy/NS	All studies	3	1.5 (0.7–3)	35
			Moderate bias	1	0.8 (0.3–2.3)	–
			Serious bias	2	2 (0.8–4.7)	26
Bathing in ponds	H5N1	–	All studies	3	3.1 (1.5–6.4)	5
			Moderate bias	2	2.5 (1.1–5.3)	0
			Serious bias	1	11.3 (1.3–102.2)	–
Using outdoor water source	H5N1	–	All studies	3	2.5 (0.5–12.1)	81
			Moderate bias	2	1.8 (0.3–12.6)	89
			Serious bias	1	6.8 (0.7–66.4)	–
Touching (unspecified practice)	H5N1	Healthy/NS	All studies	5	2.8 (1.1–7.4)	67
			Moderate bias	4	2.5 (0.9–7.5)	73
			Serious bias	1	5.8 (0.9–113.6)	–
Touching (unspecified practice)	H5N1	Sick/dead	All studies	5	4.8 (1.2–19.2)	88
			Moderate bias	3	4.7 (0.9–23.3)	88
			Serious bias	2	4.4 (0.1–195.7)	93
Touching (unspecified practice)	H7N9	Healthy/NS	All studies	6	5.5 (2.3–13.1)	71
			Moderate bias	6	5.5 (2.3–13.1)	71
			Serious bias	0	–	–
Preparing poultry	H5N1	Healthy/NS	All studies	3	3.3 (1.1–9.5)	77
			Moderate bias	1	2.2 (0.6–10.4)	–
			Serious bias	2	4.8 (0.5–45.6)	88

CI = confidence interval; *n* = number of studies; NS = not specified; pOR = pooled odds ratio along with its 95% CI.

Supplemental Table 3

Sensitivity analysis of risk factor studies according to their location

Practice	Subtype	Health status	Sub-group	*n*	pOR (95% CI)	I^2
Poultry husbandry: cleaning	H5N1	Healthy/NS	All studies	3	1.5 (0.7–3)	35
			Households	2	1.7 (0.3–9.9)	65
			Premises	1	1.6 (0.9–2.7)	–
Touching (unspecified practice)	H5N1	Healthy/NS	All studies	5	2.8 (1.1–7.4)	67
			Households	2	1.4 (0.3–7.6)	85
			Premises	3	5.5 (2.3–13.2)	0
Touching (unspecified practice)	H5N1	Sick/dead	All studies	5	4.8 (1.2–19.2)	88
			Households	4	5.6 (0.8–39.5)	90
			Premises	1	2.6 (1.3–5.4)	–
Touching (unspecified practice)	H7N9	Healthy/NS	All studies	6	5.5 (2.3–13.1)	71
			Households	4	7.6 (2.8–20.1)	75
			Premises	2	2 (0.4–9.9)	34
Preparing poultry	H5N1	Healthy/NS	All studies	3	3.3 (1.1–9.5)	77
			Households	2	5.6 (0.8–41.5)	81
			Premises	1	1.7 (1.1–2.7)	–

CI = confidence interval; *n* = number of studies; NS = not specified; pOR = pooled odds ratio along with its 95% CI. Premises: commercial farms, live bird markets, and abattoirs.

Supplemental Table 4

Sensitivity analysis of practice prevalence studies according to their location

Practice	Setting	Subgroups	*n*	Range	I^2
Raise backyard poultry	Urban HHs	All countries	4	19–51	98
		Lao	2	40–51	81
Raise backyard poultry	Semi-urban HHs	All countries	3	34–55	91
		Lao	2	51–55	20
Raise backyard poultry	Rural HHs	All countries	15	50–96	98
		China	3	50–52	0
		Lao	3	64–86	92
		Thailand	3	68–80	74
Keep poultry inside house	HHs	All countries	6	1–87	100
		Bangladesh	2	27–80	99
		Lao	2	1–3	0
Touch when purchasing	HHs	All countries	9	5–92	100
		China (HK)	3	5–14	80
		China	3	17–71	100
		Vietnam	2	63–92	99
Slaughtering and processing poultry	HHs	All countries	11	12–85	99
		Cambodia	3	28–38	93
		China	2	12–45	100
		Thailand	2	19–59	99
Management of sick and dead poultry					
Touching	HHs	All countries	9	14–75	99
		Cambodia	5	31–75	99
		Thailand	3	28–39	73
Consumption	HHs	All countries	13	2–100	99
		Cambodia	5	14–87	99
		Lao	2	3–5	0
		Thailand	2	2–12	85
		Vietnam	2	5–5	0
Selling	HHs	All countries	7	0–100	99
		Lao	2	0–0	0
Throwing in open spaces	HHs	All countries	11	2–87	99
		Bangladesh	3	45–87	89
		Lao	2	5–16	93
		Vietnam	2	12–23	90
Burying/Incinerating	HHs	All countries	13	2–95	99
		Bangladesh	3	2–12	10
		Egypt	2	16–19	0
		Lao	2	78–87	90
		Vietnam	2	76–79	26
Preventive practices					
Handwashing	HHs	All countries	6	4–99	96
		Thailand	2	80–99	94
		Vietnam	2	96–99	0
Wearing facemask	HHs	All countries	4	0–2	0
		Thailand	2	2–2	0
Wearing aprons/changing clothes	LBMs	All countries	4	15–80	96
		Indonesia	3	15–55	81
Wearing boots	LBMs	All countries	4	30–80	92
		Indonesia	3	30–65	79

HHs = households; HK = Hong Kong; LBMs = live bird markets. Handwashing: washing hands with soap after contacts with poultry. The ranges of practice prevalence and I2 indices are presented for studies conducted within the same countries.

Supplemental Table 5

Sensitivity analysis of practice prevalence studies according to their quality score

Practice	Setting	Subgroup	*n*	Range	I^2
Raise backyard poultry	Urban HHs	All countries	4	19–51	98
		Quality 1	3	19–51	98
		Quality 2	1	51	–
Raise backyard poultry	Semi-urban HHs	All countries	3	34–55	91
		Quality 1	3	34–55	91
		Quality 2	0	–	–
Raise backyard poultry	Rural HHs	All countries	15	50–96	98
		Quality 1	6	52–90	98.1
		Quality 2	9	50–96	97.9
Keep poultry inside house	HHs	All countries	6	1–87	100
		Quality 1	4	1–87	100
		Quality 2	2	14–80	99
Visit LBMs	Urban HHs	All countries	3	33–81	99.8
		Quality 1	1	81	–
		Quality 2	2	33–38	91.2
Visit LBMs	Semi-urban HHs	All countries	1	77	–
		Quality 1	1	77	–
		Quality 2			
Visit LBMs	Rural HHs	All countries	3	7–9	0
		Quality 1	3	7–9	0
		Quality 2			
Touch when purchasing	HHs	All countries	9	5–92	100
		Quality 1	7	5–92	99.7
		Quality 2	2	17–71	99.8
Slaughtering and processing poultry	HHs	All countries	11	12–85	99
		Quality 1	9	19–85	99.4
		Quality 2	2	12–85	99.6
Management of sick and dead poultry					
Touching	HHs	All countries	9	14–75	99
		Quality 1	5	14–75	99.4
		Quality 2	4	28–39	64.6
Consumption	HHs	All countries	13	2–100	99
		Quality 1	6	2–45	98.5
		Quality 2	7	2–100	99.4
Selling	HHs	All countries	7	0–100	99
		Quality 1	4	0–26	95.7
		Quality 2	3	72–100	83.1
Throwing in open spaces	HHs	All countries	11	2–87	99
		Quality 1	6	2–45	97.9
		Quality 2	5	48–87	87.8
Burying/Incinerating	HHs	All countries	13	2–95	99
		Quality 1	6	2–95	99.4
		Quality 2	7	10–76	98
Preventive practices					
Handwashing	HHs	All countries	6	4–99	96
		Quality 1	1	80	–
		Quality 2	5	4–99	96.3
Wearing gloves	HHs	All countries	3	1–2	0
		Quality 1	0	–	–
		Quality 2	3	1–2	0
Wearing facemask	HHs	All countries	4	0–2	0
		Quality 1	1	2	–
		Quality 2	3	0–2	0
Wearing aprons/changing clothes	HHs	All countries	2	0–5	0
		Quality 1	0	–	–
		Quality 2	2	0–5	0
Wearing boots	HHs	All countries	1	6	–
		Quality 1	0	–	–
		Quality 2	1	6	–
Rinsing/washing equipment	HHs	All countries	2	33–99	97.7
		Quality 1	0	–	–
		Quality 2	2	33–99	97.7

HHs = households; LBMs = live bird markets. Handwashing: washing hands with soap after contacts with poultry. The ranges of practice prevalence and I2 indices are presented for studies from the same quality group.

Supplemental Table 6

Risky practices of which their association with AIV infection was assessed in ecological studies

Exposure	Measure of association (95% CI)	Author (year)
Poultry density (1,000/km^2)	RR = 1 (0.8–1.1), $P = 0.59$	Yupiana (2010)
The number of poultry outbreaks	RR = 1.3 (1.1–1.7), $P = 0.02$	Yupiana (2010)
LBM density	BRT weight > 5	Fang (2013)
	OR = 1.1 (1–1.1), $P < 0.001$	Fuller (2014)
LBM closure	Reduction in daily incidence<br>Shanghai: 99% (93–100%)<br>Hangzhou: 99% (92–100%)<br>Huzhou: 97% (68–100%)<br>Nanjing: 97% (81–100%)	Yu (2014)
	Reduction in daily incidence<br>Overall estimate: 97% (89–100%)	Wu (2014)

BRT = boosted regression tree; CI = confidence interval; LBM = live bird market; OR = odds ratio; RR = relative risk.

Supplemental Table 7

Additional variables assessing the association between AIV infection and proximity between poultry and humans, size of susceptible poultry populations, frequency of exposure, and premise type

Exposure category	Exposure	Subtype	Non-adj OR (95% CI)	Adj OR (95% CI)	Author (year)
Raising poultry at home: cages inside/outside the house	Poultry cages inside the house vs. no poultry	H5N1	9.7 (1.8–53.3)		Zhou (2009)
	Poultry cages outside the house vs. no poultry	H5N1	3.7(0.9–15.3)		Zhou (2009)
Raising poultry at home: vaccinated flocks	Poultry H5 vaccination coverage < 80% vs. no poultry	H5N1	7.1 (1.6–31.6)		Zhou (2009)
	Poultry H5 vaccination coverage > 80% vs. no poultry	H5N1	4.0 (0.9–17.9)		Zhou (2009)
	Waterfowl H5 vaccination coverage < 80% vs. no waterfowl	H5N1	8.4 (1.6–45.1)		Zhou (2009)
	Waterfowl H5 vaccination coverage > 80% vs. no waterfowl	H5N1	2.4 (0.5–11.2)		Zhou (2009)
Raising poultry at home: flock size	Poultry number	H5N1	2.4 (1–5.7)		Huo (2012)
Working with poultry: flock size	Number of poultry bred > 1,000	H5N1		3.8 (1.7–8.7)	Li (2013)
Visiting LBM: visit frequency	1–5 visits within 2 weeks before illness vs. no visits	H5N1	2.8 (0.9–8.1)		Zhou (2009)
	6–10 visits within 2 weeks before illness vs. no visits	H5N1	7.6 (1.1–53.7)		Zhou (2009)
	> 10 visits within 2 weeks before illness vs. no visits	H5N1	5.8 (1.2–28.6)		Zhou (2009)
	1–9 visits vs. no visits	H7N9	3.4 (1.2–9.3)	3.8 (1.3–10.8)	Li (2014)
	> 10 visits vs. no visits	H7N9	5.6 (1.2–26.9)	10.6 (1.9–60.7)	Li (2014)
	1–5 visits vs. no visits	H7N9	1.5 (0.4–5.5)		Ai (2013)
	6–9 visits vs. no visits	H7N9	0.8 (0.1–7.4)		Ai (2013)
	> 10 visits vs. no visits	H7N9	8.8 (2.2–35.1)		Ai (2013)
Working with poultry: premise type	Farm type: layer vs. broiler	H7		0.8 (0.3–2.5)	Ahad (2014)
	Farm type: breeder vs. broiler	H7		3.8 (1.4–10.1)	Ahad (2014)
	Farm type: layer vs. broiler duck	H9	6.4 (1.7–23.6)		Yang (2012)
	Work in retail vs. wholesale/ farm/other premises	H5N1	2.7 (1.5–4.9)		Bridges (2002)
Working with poultry: type of activity	Chicken butcher vs. chicken keeper	H9	3.4 (0.8–14.5)		Yu (2013)
Poultry species	Raise chickens only (no waterfowls)	H5N1	2.6 (0.6–12.1)		Zhou (2009)
	Raise waterfowl	H5N1	6.4 (1.6–26.3)		Zhou (2009)
	Exposed to geese	H5N1	3.6 (1.2–10.2)	3.1 (0.9–10.4)	Gomaa (2015)
	Exposed to turkeys	H5N1	3.8 (1.3–11.3)	2.7 (0.8–9.5)	Gomaa (2015)
	Exposed to ducks	H9	4.7 (1.1–19.7)	5.7 (1.3–25.2)	Gomaa (2015)
	Duck keeper vs. chicken keeper	H9	1.1 (0.3–4)		Yu (2013)

adj OR = odds ratios adjusted for other exposures, along with 95% confidence interval; CI = confidence interval; LBM = live bird market; non-adj OR = odds ratios non-adjusted for other exposures, along with 95% confidence interval.

Supplemental Table 8

Characteristics of studies exploring the prevalence of poultry exposure practices

Reference	Country	Year	Study population	*n*	Sampling	Data	Study level	Q
Leslie (2008)[27]	Afghanistan	2007	Residents	304	prob.	quest.	National	1
Vong (2006)[28]	Cambodia	2005	Residents	155	non-prob.	quest.	District	2
Liao (2009)[29]	China	2006	Residents	1550	prob.	quest.	Province	1
Wang (2014)[30]	China	2013	Residents	3731	non-prob.	quest.	National	2
Radwan (2011)[31]	Egypt	2011	Residents	150	non-prob.	quest.	District	2
Santhia (2009)[32]	Indonesia	2005	Residents	291	prob.	quest.	Province	1
Barennes (2010)[33]	Lao	2007	Residents	1098	prob.	quest.	National	1
Wilson (2007)[34]	Lao	2007	Residents	ns	non-prob.	quest.	National	2
Maton (2007)[35]	Thailand	2005	Residents	784	prob.	quest.	Province	1
Somrongthong (2012)[36]	Thailand	2008	Residents	968	non-prob.	quest.	Province	2
Edirne (2011)[37]	Turkey	2007–2008	Residents	1046	prob.	quest.	Province	1
Rimi (2014)[38]	Bangladesh	2009	Residents	252	non-prob.	obs./int.	District	2
Cavailler (2010)[23]	Cambodia	2007	Residents	700	non-prob.	quest.	District	2
Peng (2014)[39]	China	2007	Residents	4950	prob.	quest.	District	2
Gai (2008)[40]	China	2007–2008	Residents	1379	prob.	quest.	National	1
Fielding (2005)[41]	China (HK)	2004	Residents	986	prob.	quest.	National	1
Fielding (2007)[42]	China (HK)	2005–2006	Residents	1760	prob.	quest.	National	1
Fielding (2014)[43]	China (HK)	2010	Residents	461	prob.	quest.	National	1
Barennes (2007)[44]	Lao	2006	Residents	461	prob.	quest.	National	1
Olsen (2005)[45]	Thailand	2004	Residents	200	prob.	quest.	District	2
Dejpichai (2009)[46]	Thailand	2005	Residents	131	non-prob.	quest.	National	2
Liao (2014)[47]	Thailand	2006	Residents	907	prob.	quest.	Province	1
Fielding (2007)[42]	Vietnam	2005–2006	Residents	1988	prob.	quest.	National	1
Liao (2014)[47]	Vietnam	2006	Residents	994	prob.	quest.	National	1
Manabe (2011)[48]	Vietnam	2009	Residents	418	prob.	quest.	District	2
Manabe (2012)[49]	Vietnam	2011	Residents	322	prob.	quest.	District	2
Sultana (2012a)[50]	Bangladesh	2008	Res. with birds	106	non-prob.	obs./int.	District	2
Sultana (2012b)[51]	Bangladesh	2008	Resident with birds	40	non-prob.	obs./int.	District	2
Khan (2012)[52]	Bangladesh	2011	Resident with birds	300	prob.	quest.	National	1
Ly (2007)[53]	Cambodia	2006	Resident with birds	269	prob.	quest.	National	1
Van Kerkhove (2009)[54]	Cambodia	2006	Resident with birds	452	prob.	quest.	National	1
Van Kerkhove (2008)[55]	Cambodia	2007	Resident with birds	3600	prob.	quest.	National	1
Van Kerkhove (2009)[54]	Cambodia	2007	Resident with birds	800	prob.	quest.	National	1
Khun (2012)[56]	Cambodia	2009	Resident with birds	246	prob.	quest.	District	2
Negro-Calduch (2013)[57]	Egypt	2009–2010	Resident with birds	102	non-prob.	quest.	Province	2
Kayali (2011)[58]	Lebanon	2010	Resident with birds	200	non-prob.	quest.	National	2
Nasreen (2013)[59]	Bangladesh	2009	Farm workers	212	non-prob.	quest.	National	2
Yu (2013)[13]	China	2009–2010	Farm workers	305	non-prob.	quest.	Province	2
Negro-Calduch (2013)[57]	Egypt	2009–2010	Farm workers	124	non-prob.	quest.	Province	2
Robert (2010)	Indonesia	2007	Farm workers	495	non-prob.	quest.	District	2
Neupane (2012)	Nepal	2009	Farm workers	96	prob.	quest.	District	2
Sarker (2011)	Bangladesh	2008–2009	Market workers	318	non-prob.	quest.	District	2
Ma (2014)	China	2013	Market workers	306	prob.	quest.	Province	1
Kumar (2013)	India	2008	Market workers	207	non-prob.	quest.	District	2
Santhia (2009)	Indonesia	2005	Market workers	87	non-prob.	quest.	District	2
Samaan (2012)[65]	Indonesia	2008	Market workers	34	non-prob.	quest.	District	2
Samaan (2011)[66]	Indonesia	2010	Market workers	37	non-prob.	quest.	District	2
Sutanto (2013)[52]	Indonesia	2012	Market workers	100	prob.	quest.	District	2
Kuo (2011)[67]	Taiwan	2009–2010	Market workers	177	non-prob.	quest.	District	2
Li (2013)[24]	China	2010–2012	Poultry workers	1169	non-prob.	quest.	Province	2
Yang (2012)[18]	China	2011	Poultry workers	1741	prob.	quest.	Province	1

HK = Hong Kong; obs./int. = observations and/or in-depth interviews; Q = quality grade; quest.: standardized questionnaires. National: several provinces are included in the study; district: the study was conducted at the level of a district, or below. Sampling refers to the sampling strategy, probabilistic (prob.) or non-probabilistic (non-prob.) sampling.

Supplemental Table 9

Prevalence of poultry exposure practices

Practice/country	Population/year	*n*	p	CI	Author (year)
Bury/incinerate dead birds/	Household/				
Bangladesh		40	12	4–27	Sultana (2012b)
Bangladesh	2009	30	10	2–27	Rimi (2014)
Bangladesh	2011	300	2	1–4	Khan (2012)
Cambodia	2009	246	72	65–77	Khun (2012)
China	2007	127	38	29–47	Peng (2014)
Egypt	2009–2010	102	19	12–28	Negro-Calduch (2013)
Egypt	2011	127	16	10–23	Radwan (2011)
Indonesia	2005	291	80	75–85	Santhia (2009)
Lao	2006	399	78	73–82	Barennes (2007)
Lao	2007	1098	87	85–89	Barennes (2010)
Thailand	2006	907	95	93–96	Liao (2014)
Vietnam	2006	994	79	76–81	Liao (2014)
Vietnam	2009	418	76	71–80	Manabe (2011)
Bury/incinerate dead birds/	Farm/				
Egypt	2009–2010	124	30	22–39	Negro-Calduch (2013)
Nepal	2009	96	95	88–98	Neupane (2012)
Consuming dead birds/	Household/				
Bangladesh	2008	40	100	91–100	Sultana (2012b)
Cambodia	2006	269	45	39–51	Ly (2007)
Cambodia	2006	452	45	40–50	Van Kerkhove (2009)
Cambodia	2007	700	56	52–60	Cavailler (2010)
Cambodia	2007	800	14	11–16	Van Kerkhove (2009)
Cambodia	2009	246	87	83–91	Khun (2012)
China	2007	127	3	1–8	Peng (2014)
Lao	2006	399	2	1–5	Barennes (2007)
Lao	2007	1098	5	4–6	Barennes (2010)
Thailand	2004	200	12	8–17	Olsen (2005)
Thailand	2008	968	2	1–3	Somrongthong (2012)
Vietnam	2005–2006	253	5	3–9	Fielding (2007)
Vietnam	2009	418	5	3–8	Manabe (2011)
Consuming dead birds/	Market/				
Bangladesh	2008–2009	318	16	12–21	Sarker (2011)
Keep poultry inside house/	Household/				
Bangladesh	2008	106	80	71–87	Sultana (2012a)
Bangladesh	2011	300	27	22–32	Khan (2012)
Cambodia	2007	2401	87	85–88	Van Kerkhove (2008)
Egypt	2011	118	14	8–21	Radwan (2011)
Lao	2006	428	3	1–5	Barennes (2007)
Lao	2007	1098	1	0–1	Barennes (2010)
Raise backyard poultry/	Household/				
Turkey	2007–2008	1046	66	63–69	Erdine (2011)
Vietnam	2005–2006	1988	53	51–55	Fielding (2007)
Raise backyard poultry/	Peri-urban household/				
Lao	2006	192	50	43–58	Barennes (2007)
Lao	2007	364	55	50–61	Barennes (2010)
Raise backyard poultry/	Rural household/				
Afghanistan	2007	304	65	59–70	Leslie (2008)
Bangladesh	2009	252	81	76–86	Rimi (2014)
Cambodia	2005	155	96	92–99	Vong (2006)
China	2007	1043	51	48–54	Peng (2014)
China	2007–2008	1379	52	49–54	Gai (2008)
China	2013	1227	50	47–53	Wang (2014)
Egypt	2011	150	78	71–84	Radwan (2011)
Indonesia	2005	291	90	86–93	Santhia (2009)
Lao	2006	189	84	78–89	Barennes (2007)
Lao	2007	570	64	60–68	Barennes (2010)
Lao	2007	NA	88	85–90	Wilson (2007)
Thailand	2004	200	74	67–80	Olsen (2005)
Thailand	2005	131	68	59–76	Dejpichai (2009)
Thailand	2005	784	80	77–83	Maton (2007)
Vietnam	2011	322	92	89–95	Manabe (2012)
Raise backyard poultry/	Peri-urban household/				
China	2006	187	34	27–42	Liao (2009)
Raise backyard poultry/	Urban household/				
China	2006	1363	19	17–21	Liao (2009)
Lao	2006	461	40	36–45	Barennes (2007)
Lao	2007	164	51	43–58	Barennes (2010)

(continued)

Supplemental Table 9
Continued

Practice/country	Population/year	*n*	p	CI	Author (year)
Vietnam	2011	221	51	44–57	Manabe (2012)
Return sick birds to supplier/	Market/				
Bangladesh	2008–2009	318	9	6–13	Sarker (2011)
Return sick birds to supplier/	LBM/farm worker/				
India	2008	207	50	43–57	Kumar (2013)
Rinse/wash equipment/	Household/				
Bangladesh	2009	30	33	17–53	Rimi (2014)
Egypt	2011	127	99	96–100	Radwan (2011)
Rinse/wash equipment/	Farm/				
Nepal	2009	96	41	31–51	Neupane (2012)
Rinse/wash equipment/	Market/				
Indonesia	2008	34	38	22–56	Samaan (2012)
Indonesia	2009	29	62	42–79	Samaan (2012)
Indonesia	2010	37	19	8–35	Samaan (2011)
Indonesia	2012	100	100	96–100	Sutanto (2013)
Sell sick/dead poultry/	Household/				
Afghanistan	2007	304	26	22–32	Leslie (2008)
Bangladesh	2008	40	100	91–100	Sultana (2012b)
Cambodia	2009	246	89	85–93	Khun (2012)
Egypt	2009–2010	102	72	63–81	Negro-Calduch (2013)
Lao	2006	399	0	0–1	Barennes (2007)
Lao	2007	1098	0	0–1	Barennes (2010)
Vietnam	2005–2006	253	5	3–9	Fielding (2007)
Sell sick/dead poultry/	Farm/				
Egypt	2009–2010	124	81	73–87	Negro-Calduch (2013)
Sell sick/dead poultry	Market/				
Bangladesh	2008–2009	318	53	48–59	Sarker (2011)
India	2008	105	2	0–7	Kumar (2013)
Slaughter poultry/	Farm/				
Bangladesh	2009	212	38	32–45	Nasreen (2013)
Indonesia	2007	495	6	4–9	Robert (2010)
Slaughter poultry/	Household/				
Bangladesh	2011	300	84	80–88	Khan (2012)
Cambodia	2006	452	38	34–43	Van Kerkhove (2009)
Cambodia	2007	3600	28	27–30	Van Kerkhove (2008)
Cambodia	2007	800	36	32–39	Van Kerkhove (2009)
China	2006	1550	45	42–47	Liao (2009)
China	2013	685	12	9–14	Wang (2014)
Egypt	2011	150	85	78–90	Radwan (2011)
Indonesia	2005	841	55	52–58	Santhia (2009)
Thailand	2005	147	19	13–26	Maton (2007)
Thailand	2006	907	59	56–62	Liao (2014)
Vietnam	2006	994	85	83–87	Liao (2014)
Slaughter poultry/	Market/				
Bangladesh	2009	210	39	32–46	Nasreen (2013)
China	2013	306	87	83–91	Ma (2014)
India	2008	105	100	97–100	Kumar (2013)
Indonesia	2005	87	58	46–68	Santhia (2009)
Throw dead birds/	Household/				
Bangladesh	2008	40	48	32–64	Sultana (2012b)
Bangladesh	2009	30	87	69–96	Rimi (2014)
Bangladesh	2011	300	45	40–51	Khan (2012)
Cambodia	2009	246	75	69–80	Khun (2012)
China	2007	127	57	48–65	Peng (2014)
Egypt	2011	127	84	77–90	Radwan (2011)
Lao	2006	399	16	13–20	Barennes (2007)
Lao	2007	1098	5	3–6	Barennes (2010)
Thailand	2006	907	2	1–3	Liao (2014)
Vietnam	2005–2006	253	12	8–16	Fielding (2007)
Vietnam	2006	994	23	20–26	Liao (2014)
Throw dead birds/	LBM/Farm worker/				
India	2008	207	20	15–26	Kumar (2013)
Touch dead/sick poultry/	Household/				
Cambodia	2006	269	75	69–80	Ly (2007)
Cambodia	2006	452	75	71–79	Van Kerkhove (2009)
Cambodia	2007	700	31	28–35	Cavailler (2010)
Cambodia	2007	3600	33	31–34	Van Kerkhove (2008)
Cambodia	2007	800	42	39–46	Van Kerkhove (2009)
Thailand	2004	200	39	32–46	Olsen (2005)
Thailand	2005	131	28	21–37	Dejpichai (2009)

(continued)

Supplemental Table 9
Continued

Practice/country	Population/year	*n*	p	CI	Author (year)
Touch dead/sick poultry/	Farm/				
China	2011	1741	13	12–15	Yang (2012)
Touch dead/sick poultry/	Market/				
Indonesia	2005	87	41	31–52	Santhia (2009)
Touch dead/sick poultry/	Poultry workers/				
China	2010–2012	1169	8	7–10	Li (2013)
Touch dead/sick poultry/	Rural household/				
Indonesia	2005	841	14	12–17	Santhia (2009)
Thailand	2008	968	28	25–31	Somrongthong (2012)
Touch when purchasing/	Household/				
China	2006	1550	59	56–61	Liao (2009)
China	2007	2058	17	15–19	Peng (2014)
China	2013	685	71	67–74	Wang (2014)
China (HK)	2004	774	14	12–17	Fielding (2005)
China (HK)	2005–2006	1191	8	6–9	Fielding (2007)
China (HK)	2010	189	5	2–9	Fielding (2014)
Thailand	2006	907	63	60–66	Liao (2014)
Vietnam	2005–2006	676	63	59–67	Fielding (2007)
Vietnam	2006	994	92	90–94	Liao (2014)
Visit LBMs/	Household/				
China (HK)	2004	986	78	76–81	Fielding (2005)
China (HK)	2005–2006	1760	71	69–73	Fielding (2007)
China (HK)	2010	461	41	36–46	Fielding (2014)
Vietnam	2005–2006	1988	34	32–36	Fielding (2007)
Visit LBMs/	Rural household/				
Cambodia	2006	452	9	7–12	Van Kerkhove (2009)
Cambodia	2007	3600	7	6–8	Van Kerkhove (2008)
Cambodia	2007	800	8	6–10	Van Kerkhove (2009)
Visit LBMs/	Peri-urban household/				
China	2006	187	77	70–83	Liao (2009)
Visit LBMs/	Urban household/				
China	2006	1363	81	79–83	Liao (2009)
China	2007	2058	38	36–40	Peng (2014)
China	2013	2504	33	31–35	Wang (2014)
Wash hands with soap/	Household/				
Bangladesh	2009	30	4	0–17	Rimi (2014)
Egypt	2011	118	98	94–100	Radwan (2011)
Thailand	2005	147	80	73–86	Maton (2007)
Thailand	2008	691	99	98–100	Somrongthong (2012)
Vietnam	2009	418	96	94–98	Manabe (2011)
Vietnam	2011	322	99	97–100	Manabe (2012)
Wash hands with soap/	Market/				
Bangladesh	2008–2009	318	68	63–73	Sarker (2011)
Wear aprons/change clothes/	Household/				
Egypt	2011	118	5	2–11	Radwan (2011)
Lebanon	2010	128	0	0–3	Kayali (2011)
Wear aprons/change clothes/	Farm/				
Bangladesh	2009	212	34	28–41	Nasreen (2013)
Lebanon	2010	72	4	1–12	Kayali (2011)
Nepal	2009	96	3	1–9	Neupane (2012)
China	2009–2010	305	89	85–92	Yu (2013)
Wear aprons/change clothes/	Market/				
China	2013	306	80	75–84	Ma (2014)
Indonesia	2008	34	15	5–31	Samaan (2012)
Indonesia	2009	29	55	36–74	Samaan (2012)
Indonesia	2010	37	30	16–47	Samaan (2011)
Wear boots/	Household/	128	6	3–12	Kayali (2011)
Lebanon	2010				
Wear boots/	Farm/				
Bangladesh	2009	212	16	11–22	Nasreen (2013)
Lebanon	2010	72	15	8–26	Kayali (2011)
Nepal	2009	96	7	3–14	Neupane (2012)
Wear boots/	Market/				
China	2013	306	80	75–84	Ma (2014)
Indonesia	2008	34	65	46–80	Samaan (2012)
Indonesia	2009	29	55	36–74	Samaan (2012)
Indonesia	2010	37	30	16–47	Samaan (2011)
Wear facemask/	Household/				
Egypt	2011	127	2	0–6	Radwan (2011)
Lebanon	2010	128	0	0–3	Kayali (2011)

(continued)

Supplemental Table 9
Continued

Practice/country	Population/year	*n*	p	CI	Author (year)
Thailand	2005	147	2	0–6	Maton (2007)
Thailand	2008	691	2	1–3	Somrongthong (2012)
Wear facemask/	Farm/				
Bangladesh	2009	212	66	59–72	Nasreen (2013)
Indonesia	2007	495	14	11–18	Robert (2010)
Lebanon	2010	72	6	2–14	Kayali (2011)
Nepal	2009	96	27	19–37	Neupane (2012)
China	2009–2010	305	46	40–52	Yu (2013)
China	2011	1741	42	40–44	Yang (2012)
Wear facemask/	Market/				
Bangladesh	2008–2009	318	17	13–21	Sarker (2011)
China	2013	306	20	16–25	Ma (2014)
Indonesia	2012	100	13	7–21	Sutanto (2013)
Taiwan	2009–2010	177	45	38–53	Kuo (2011)
Wear gloves/	Household/				
Egypt	2011	127	2	0–6	Radwan (2011)
Lebanon	2010	128	2	0–6	Kayali (2011)
Thailand	2008	691	1	0–2	Somrongthong (2012)
Wear gloves/	Farm/				
Bangladesh	2009	212	27	21–33	Nasreen (2013)
Indonesia	2007	495	10	8–13	Robert (2010)
Lebanon	2010	72	1	0–7	Kayali (2011)
Nepal	2009	96	30	21–40	Neupane (2012)
China	2009–2010	305	54	48–60	Yu (2013)
China	2011	1741	47	45–49	Yang (2012)
Wear gloves/	Market/				
China	2013	306	60	54–66	Ma (2014)

CI = confidence interval; HK = Hong Kong; LBM = live bird market; *n* = sample size; p = proportion of respondents adopting the practice. Wash hands with soap: handwashing after contacts with poultry; throw dead birds: throw dead birds in open space; visit LBMs: visit to LBMs to purchase poultry.

Supplemental Table 10

Identified rationales by authors based on responses to interviews and observation for poultry exposure practices in reviewed practice prevalence studies

Practice	Summary of rationale	Setting	Country	Author (year)
To keep poultry inside the bedroom	They are concerned about the security of their poultry. They want to protect them from jungle cats, foxes, and thieves.	HH	Bangladesh	Sultana (2012)
To keep sick poultry inside the bedroom	Sick poultry are kept under a basket in the yard during the daytime to separate them from their healthy poultry, and under the bed at night to observe if the poultry were about to die.	HH	Bangladesh	Sultana (2012b)
To consume sick poultry	They slaughter and consume the sick poultry if they thought that the poultry would not recover because Islam prohibits eating animals that die of natural causes	HH	Bangladesh	Sultana (2012b)
	Unable to sell sick poultry, they consume them, in accordance with the Islamic principles. They slaughter their healthy poultry in fear of losing them, when hearing about high mortality in neighboring flocks	HH	Bangladesh	Rimi (2014)
Cleaning ground after slaughter	First because they want to avoid disease among poultry, second because blood looks bad, and third because stepping on blood might cause harm to others, pregnant women in particular	HH	Bangladesh	Rimi (2014)
Buying live poultry	Traditional habit, lower meat quality of chilled or frozen meat	Market	China	Ma (2014)
Risky practices	These practices are traditional. Traditional practices are explained as a matter of preference.	HH	Turkey	Erdine (2011)
	They relate to the perception that people and poultry are not at risk. Most people were aware of avian influenza, but estimated the risks associated with poultry handling to be negligible.	Market	Lao	Barennes (2007)
No preventive practices	Farmers cannot afford their implementation	HH	Lao	Barennes (2010)
	Lack of willingness, economic dependence on limited resources, and low living standards.	HH	Turkey	Erdine (2011)
	Villagers do not believe that AIVs can transmit to humans. They consider the chance of an adverse outcome from poultry exposure to be quite low compared with the adverse outcome of worse poverty, and are therefore less likely to change their behavior to decrease the frequency of poultry–human interactions.	HH	Bangladesh	Sultana (2012)
	Absence of supporting legislation, financial constraints, time, and space constraints	HH/Farm	Egypt	Negro-Calduch (2013)
	Fear of losing costumers, may reduce sales, may increase costs.	Market	Indonesia	Sutanto (2013)

AIV = avian influenza virus; HH = household.

Supplemental Table 11

Examples of rationales discussed as post hoc hypotheses by authors of practice prevalence and risk factor studies

Practice	Summary of rationale	Setting	Country	Author (year)
Buying live poultry	They prefer live poultry to pre-killed for family consumption. Poultry slaughtered immediately before cooking are traditionally believed to be fresher, better in flavor, more nutritious and less likely to be contaminated	HH	Vietnam	Liao (2014)
Hygiene measures in markets/ preventive measures	Resistance due to "risk fatigue" from repeated wet market-related outbreaks combined with economic concerns. Effective preventive measures would threaten economic security among traders with low education and few employment alternatives	Market	China	Ma (2014)
Rear backyard poultry	Poultry prices have increased, making poultry consumption a privilege of the wealthier Vietnamese. This encourages the continuation of backyard farming	HH	Vietnam	Liao (2014)
Consumption of sick and dead birds	Poultry prices have increased, making poultry consumption a privilege of the wealthier Vietnamese. This encourages the continuation of backyard farming, including the full use of sick/dead birds	HH	Vietnam	Liao (2014)
Slaughtering poultry	According to Buddhist principles, killing is considered to have karmic consequences. Thai people are therefore less likely to slaughter the poultry themselves	HH	Thailand	Liao (2014)
Touching poultry before purchase	The reason for touching and feeling the poultry before buying relates to the consumer tradition, relying on their own judgment on the quality and safety of the poultry	HH	Vietnam/Thailand	Liao (2014)
Risky practices	Farmers' inadequate knowledge, awareness, and information about zoonosis	HH	Bangladesh	Khan (2012)
	People have believed that people could never get infected with HPAI by engaging in risky practices. The study notes that this may be strongly related to poverty and illiteracy	HH	Cambodia	Khun (2012)

HH = household.

SUPPLEMENTAL TABLE 12

Risky and protective practices of which their association with AIV infection was assessed in the reviewed risk factor studies

Exposure	H.	L.	Non-adj OR (95% CI)	Adj OR (95% CI)	Author (year)
Poultry in the vicinity: neighbors raising poultry					
Live poultry in neighborhood	NS		1.1 (0.2–6.6), $P = 0.81$		Dinh (2006)
Poultry in the vicinity: poultry farms in the vicinity					
Poultry farms in the vicinity	NS		13 (2.4–133)	42 (2.3–1000)	He (2014)
Poultry in the vicinity: neighbors raising poultry					
Neighbors raising poultry at home	NS		6.4 (2.9–15)		He (2014)
Raising poultry or pigeons in the neighborhood	NS		0.9 (0.4–2.2), $P = 0.82$		Li (2014)
Sick or dead poultry in the neighborhood	Sick		3.9 (1.0–55.7), $P = 0.05$		Dinh (2006)
In location with poultry: attending cockfight					
Attended cockfight	NS		1.5 (0.3–5.5), $P = 0.47$		Cavailler (2010)
In location with poultry: visiting LBM, neighbors raising poultry					
Visiting markets, and poultry raised in the neighborhood	NS		6.9 (1.9–25.2), $P = 0.003$	4.2 (0.9–19.6), $P = 0.064$	Ai (2013)
In location with poultry					
In location with poultry	NS		2.3 (1.0–5.3), $P = 0.06$		Liu (2014)
In location with poultry: raising poultry at home					
Raise poultry	NS	H	0.7 (0.1–6.7), $P = 0.75$		Ai (2013)
Being < 1 m away from dead poultry	Sick	H	13 (1.8–96.3)		Areechokchai (2006)
Live poultry in household	NS	H	3 (0.9–10), $P = 0.1$		Dinh (2006)
Sick or dead poultry in household	Sick	H	7.4 (2.7–59), $P < 0.001$	4.9 (1.2–20.2), $P = 0.03$	Dinh (2006)
Raising poultry at home	NS	H	9 (2.6–39)		He (2014)
Indirect contact with poultry at home	NS	H	4.4 (1.1–18.2), P 0.04		Li (2014)
Exposure to backyard poultry at home (raise)	NS	H	2.4 (1.1–5.6), $P = 0.04$	1.5 (0.5–4.4), $P = 0.48$	Liu (2014)
Sick or dying backyard poultry in month before symptoms	Sick	H	9.4 (2.5–36.1), $P < 0.01$	9.8 (2.2–43.2) $P < 0.01$	Liu (2014)
Live birds in home	NS	H	1.4 (0.3–6.4), $P < 0.9$		Mounts (1999)
Raise backyard poultry	NS	H	4.5 (1.1–17.5) $P = 0.03$		Zhou (2009)
Indirect contact with sick and/or dead poultry	Sick	NS	11.3 (2.2–58.5), $P = 0.004$	57 (4.3–746), $P = 0.002$	Zhou (2009)
Raising poultry at home: cages inside the house					
Poultry cages inside the house vs. no poultry	NS	H	9.7 (1.8–53.3) $P = 0.009$		Zhou (2009)
Raising poultry at home: cages outside the house					
Poultry cages outside the house vs. no poultry	NS	H	3.7 (0.9–15.3) $P = 0.07$		Zhou (2009)
Raising poultry at home: vaccinated flocks					
Poultry H5 vaccination coverage < 80% vs. no poultry	NS	H	7.1 (1.6–31.6), $P = 0.01$		Zhou (2009)
Poultry H5 vaccination coverage > 80% vs. no poultry	NS	H	4.0 (0.9–17.9), $P = 0.07$		Zhou (2009)
Waterfowl H5 vaccination coverage < 80% vs. no waterfowl	NS	H	8.4 (1.6–45.1), $P = 0.01$		Zhou (2009)
Waterfowl H5 vaccination coverage > 80% vs. no waterfowl	NS	H	2.4 (0.5–11.2), $P = 0.26$		Zhou (2009)
Raising poultry at home: flock size					
Poultry number	NS	H	2.4 (1–5.7), $P = 0.03$		Huo (2012)
In location with poultry: Poultry species					
Raise chickens only (no waterfowls)	NS	H	2.6 (0.6–12.1)		Zhou (2009)
Raise waterfowl	NS	H	6.4 (1.6–26.3)		Zhou (2009)
Exposed to geese	NS	NS	3.6 (1.2–10.2)	3.1 (0.9–10.4)	Gomaa (2015)
Exposed to turkeys	NS	NS	3.8 (1.3–11.3)	2.7 (0.8–9.5)	Gomaa (2015)
Exposed to ducks	NS	NS	4.7 (1.1–19.7)	5.7 (1.3–25.2)	Gomaa (2015)

(continued)

Supplemental Table 12
Continued

Exposure	H.	L.	Non-adj OR (95% CI)	Adj OR (95% CI)	Author (year)
Duck keeper vs. chicken keeper	NS	F	1.1 (0.3–4)		Yu (2013)
In location with poultry: working on a poultry premise					
Occupational poultry exposure	NS	P	13.1 (1.4–125.4), *P* = 0.03		Zhou (2009)
> 10% Mortality among poultry	Sick	P	2.2 (1.3–3.7)		Bridges (2002)
Occurrence of sick/dead ducks at worksite	Sick	F	1.1 (0.2–4.9), *P* = 0.94		Yang (2012)
Working with poultry: premise type					
Farm type: layer vs. broiler	NS	F		0.8 (0.3–2.5)	Ahad (2014)
Farm type: breeder vs. broiler	NS	F		3.8 (1.4–10.1)	Ahad (2014)
Farm type: layer vs. broiler duck	NS	F	6.4 (1.7–23.6), *P* = 0.006		Yang (2012)
Work in retail vs. wholesale/farm/other poultry industry	NS	M	2.7 (1.5–4.9)		Bridges (2002)
Working with poultry: flock size					
Number of poultry bred > 1,000	NS	P		3.8 (1.7–8.7), *P* = 0.001	Li (2013)
In location with poultry: visiting LBM					
Visiting a wet poultry market	NS	M	3.1 (1.2–7.9), *P* = 0.02	15.4 (3.0–80.2), *P* = 0.001	Zhou (2009)
Visiting a wet poultry market (computed)	NS	M	3.8 (1.5–9.8)		Li (2014)
Visit LBM	NS	M	10.4 (4.9–22.0), *P* < 0.01	3.4 (1.8–6.7), *P* < 0.01	Liu (2014)
Purchasing of live or freshly slaughtered poultry in a market	NS	M	7.9 (3–23)	4.9 (1.2–24)	He (2014)
Visited LBM	NS	M	3.1 (1.1–8.3), *P* = 0.03		Ai (2013)
Exposure to poultry in market	NS	M	4.5 (1.2–21.7), *P* = 0.05		Mounts (1999)
In location with poultry: visiting informal LBM					
Visiting a temporary roadside poultry vendor	NS	M	7 (1.2–40.6), *P* = 0.03		Li (2014)
Visiting LBM: visit frequency					
1–5 visits within 2 weeks before illness vs. no visits	NS	M	2.8 (0.9–8.1), *P* = 0.06		Zhou (2009)
6–10 visits within 2 weeks before illness vs. no visits	NS	M	7.6 (1.1–53.7), *P* = 0.04		Zhou (2009)
> 10 visits within 2 weeks before illness vs. no visits	NS	M	5.8 (1.2–28.6), *P* = 0.03		Zhou (2009)
Visiting a live poultry market: 1–9 times vs. no	NS	M	3.4 (1.2–9.3), *P* = 0.02	3.8 (1.3–10.8), *P* = 0.01	Li (2014)
Visiting a live poultry market: > 10 times vs. no	NS	M	5.6 (1.2–26.9), *P* = 0.03	10.6 (1.9–60.7), *P* = 0.008	Li (2014)
Market: 1–5 visits vs. no visits	NS	M	1.5 (0.4–5.5), *P* = 0.55		Ai (2013)
Market: 6–9 visits vs. no visits	NS	M	0.8 (0.1–7.4), *P* = 0.82		Ai (2013)
Market: > 10 visits vs. no visits	NS	M	8.8 (2.2–35.1), *P* = 0.002		Ai (2013)
Poultry husbandry: cleaning					
Removed/cleaned feces from poultry areas	NS	H	5 (0.7–36.3), *P* = 0.09		Vong (2009)
Cleaning poultry stalls (job duty)	NS	P	1.6 (0.9–2.7)		Bridges (2002)
Cleaning henhouse	NS	M	2.6 (0.8–8), *P* = 0.1		Wang (2014)
Cleaned poultry stalls	NS	H	0.8 (0.3–2.3), *P* = 0.71		Cavailler (2010)
Poultry husbandry: collecting eggs					
Touched/collected eggs	NS	H	2.5 (0.2–26), *P* = 0.44		Vong (2009)
Collecting eggs (job duty)	NS	P	1.2 (0.6–2.2)		Bridges (2002)
Poultry husbandry: feeding					
Fed poultry	NS	H	0.6 (0–19.5), *P* = 0.64		Vong (2009)
Feeding poultry (job duty)	NS	P	2.4 (1.4–4.1)		Bridges (2002)
Feeding	NS	M	1.8 (0.3–10), *P* = 0.53		Wang (2014)

(continued)

Supplemental Table 12

Continued

Exposure	H.	L.	Non-adj OR (95% CI)	Adj OR (95% CI)	Author (year)
Selling poultry: handling money					
Handling money (job duty)	NS	P	1.6 (1.0–2.5)		Bridges (2002)
Processing, preparing poultry: helped/witnessed					
Helped prepare or cook sick or dead poultry	Sick	H	2.6 (0.8–8.7), $P = 0.1$		Dinh (2006)
Witnessed poultry slaughtering at market	NS	M	5 (1.7–14.9), $P = 0.004$		Zhou (2009)
Processing, preparing poultry: storing poultry products					
Storing products of sick or dead poultry in house	Sick	H	9.3 (2.1–41.3)		Areechokchai (2006)
Bathing in pond					
Swim/bathe in ponds	NS	H	11.3 (1.3–102.2), $P = 0.03$		Vong (2009)
Swam/bathed in pond	NS	H	2.5 (1–6.5), $P = 0.05$	3 (1.1–8.4), $P = 0.04$	Cavailler (2010)
Wading in ponds	NS	H	2.4 (0.4–19.2), $P = 0.47$		Dinh (2006)
Using outdoor water sources					
Use pond as source of water	NS	H	6.8 (0.7–66.4), $P = 0.08$		Vong (2009)
No indoor water source (use outdoor sources)	NS	H	5 (1.3–77), $P = 0.01$	6.5 (1.2–34.8), $P = 0.03$	Dinh (2006)
Lack of indoor water supply	NS	H	0.7 (0.1–4.3), $P = 0.73$		Zhou (2009)
Touching (unspecified practice)					
Direct contact with poultry	NS	NS	13.7 (2.9–64.8), $P < 0.001$	9.1 (1.6–50.9), $P = 0.012$	Ai (2013)
Direct touching of unexpectedly dead poultry	Sick	H	29 (2.7–308)		Areechokchai (2006)
Touching poultry (job duty)	NS	P	5.8 (0.9–113.6)		Bridges (2002)
Touched/fed live poultry	NS	H	0.6 (0.2–1.6), $P = 0.34$		Cavailler (2010)
Touched sick poultry	Sick	H	1.4 (0.6–3.8), $P = 0.46$		Cavailler (2010)
Tending to home-raised poultry	NS	H	19 (4–182)	9.9 (0.4–318)	He (2014)
Direct or close contact with poultry	NS	P	5.9 (1.8–19), $P < 0.001$	5.2 (1.5–17.7), $P = 0.008$	Li (2013)
Direct or close contact with sick/dead poultry	Sick	P	2.6 (1.3–5.4), $P < 0.02$		Li (2013)
Occupational direct contact with poultry	NS	P	6.4 (0.6–74.2), $P = 0.14$		Li (2014)
Raising poultry or pigeons at home (direct contact)	NS	H	3.2 (0.6–17.2), $P = 0.16$		Li (2014)
Touching live poultry with bare hands in a market	NS	M	1.1 (0.6–2.2), $P = 0.74$		Li (2014)
Poultry contact (feeding, capturing, cleaning, etc.)	NS	NS	3.6 (1.7–7.3), $P < 0.01$		Liu (2014)
Touched sick and/or dead poultry with bare hands	Sick	H	0.6 (0.1–4.5), $P = 0.61$		Vong (2009)
Handling ducks with wounds on hands	NS	F	6.3 (2–20.1), $P = 0.002$	4.1 (1.3–13.6), $P = 0.02$	Yang (2012)
Close contact with sick/dead ducks	Sick	F	0.6 (0.1–4.6), $P = 0.61$		Yang (2012)
Direct contact with healthy-appearing poultry	H	NS	3.3 (1.0–10.4), $P = 0.04$		Zhou (2009)
Direct contact with live poultry at the market	NS	M	4.6 (0.4–51.9), $P = 0.22$		Zhou (2009)
Direct contact with sick and/or dead poultry	Sick	NS	34.7 (4.3–277), $P < 0.001$	507 (16–16320), $P < 0.001$	Zhou (2009)
Direct contacts: butchers vs. keepers					
Occupational exposure: butchers vs. keepers	NS	P	3.4 (0.8–14.5), $P = 0.09$		Yu (2013)
Contact with poultry from home vs. markets					
Indirect or direct contact with poultry (backyard or from markets) at home	NS	H	3.9 (2–7.5), $P < 0.01$		Liu (2014)
Indirect or direct contact at home only with backyard poultry	NS	H	1.2 (0.6–2.5), $P = 0.56$		Liu (2014)
Indirect or direct contact at home with poultry traded in markets	NS	H	6.3 (2.9–13.7), $P < 0.01$		Liu (2014)

(continued)

Supplemental Table 12
Continued

Exposure	H.	L.	Non-adj OR (95% CI)	Adj OR (95% CI)	Author (year)
Processing, preparing poultry: slaughtering					
Butchering poultry (job duty)	NS	P	3.1 (1.6–5.9)		Bridges (2002)
Chopped/butchered sick poultry	Sick	H	1.6 (0.6–4.6), $P = 0.37$		Cavailler (2010)
Poultry contact during slaughtering or processing	NS	NS	3.1 (1.7–5.9), $P < 0.01$		Liu (2014)
Slaughtered and/or bled poultry	NS	H	2.5 (0.3–10.8), $P = 0.45$		Vong (2009)
Processing	NS	M	0.9 (0.3–2.4), $P = 0.85$		Wang (2014)
Processing, preparing poultry: plucking					
Plucking poultry	NS	H	14 (1.3–153)		Areechokchai (2006)
Plucked sick poultry	Sick	H	1.7 (0.6–4.8), $P = 0.33$		Cavailler (2010)
Defeathered poultry that died of illness	Sick	H	3.1 (0.3–34.9), $P = 0.35$		Vong (2009)
Processing, preparing poultry: eviscerating					
Touching poultry intestines (job duty)	NS	P	1.7 (0.9–2.9)		Bridges (2002)
Eviscerated sick poultry	Sick	H	1.2 (0.4–3.6), $P = 0.77$		Cavailler (2010)
Removed internal organs	NS	H	1.5 (0.3–8.7), $P = 0.64$		Vong (2009)
Processing, preparing poultry: preparing					
Prepared and cooked poultry or birds at home	NS	H	8.1 (2.1–31.7), $P = 0.003$		Ai (2013)
Dressing poultry	NS	H	17 (1.6–177)		Areechokchai (2006)
Preparing poultry for restaurants (job duty)	NS	P	1.7 (1.1–2.7)		Bridges (2002)
Cooked sick poultry	Sick	H	1.2 (0.4–3.6), $P = 0.78$		Cavailler (2010)
Prepared and cooked sick or dead poultry	Sick	H	31 (3.4–1150), $P < 0.001$	9 (1–82), $P = 0.05$	Dinh (2006)
Prepared and cooked healthy poultry	H	H	2.2 (0.6–10.4), $P = 0.25$		Dinh (2006)
Preparing or cooking at home	NS	H	1.4 (0.4–5), $P = 0.61$		Li (2014)
Processing, preparing poultry: washing carcass/meat					
Washed sick poultry carcasses	Sick	H	1.3 (0.5–3.8), $P = 0.59$		Cavailler (2010)
Cut/wash internal meat	NS	H	2.0 (0.27–14.9), $P = 0.49$		Vong (2009)
Consumption					
Consumed healthy-appearing poultry	H	NS	1.3 (0.4–4.2), $P = 0.61$		Zhou (2009)
Consumed poultry organs or poultry	NS	NS	0.6 (0.0–7.5), $P = 1$		Mounts (1999)
Poultry consumption	NS	NS	1.2 (0.7–2.0), $P = 0.52$	0.7 (0.4–1.4), $P = 0.28$	Liu (2014)
Ate sick poultry	Sick	H	1.3 (0.5–3.3), $P = 0.64$		Cavailler (2010)
Poultry husbandry: gathering, placing in cages					
Gathered poultry and placed in cages/poultry areas	NS	H	5.8 (1–34.1), $P = 0.05$		Vong (2009)
Poultry husbandry: transporting					
Transportation of poultry	NS	M	2.2 (0.4–12.1), $P = 0.35$		Wang (2014)
Poultry husbandry: vaccinating					
Vaccinate poultry	NS	NS	2.4 (1.2–4.8), $P = 0.009$	2.4 (1.1–5)	Gomaa (2015)
Poultry husbandry: selling					
Selling poultry	NS	M	0.7 (0.3–2), $P = 0.55$		Wang (2014)
Frequent handwashing					
Frequent handwashing		H	0.1 (0–0.6), $P = 0.008$		Ai (2013)
Handwashing > 3 times/day		H	0.5 (0.1–2.4), $P = 0.57$		Dinh (2006)
Infrequent (or no) handwashing at home		H	1.8 (0.6–6.1), $P = 0.32$		Li (2014)
Washed hands with soap after handling poultry		H	0.4 (0–3.9), $P = 0.4$		Vong (2009)
Wearing gloves					
Do not use gloves		F	2.7 (0.7–9.9), $P = 0.14$		Yang (2012)
Wearing masks					
Do not use mask		F	8.1 (1–62.6), $P = 0.05$		Yang (2012)

(continued)

Supplemental Table 12

Continued

Exposure	H.	L.	Non-adj OR (95% CI)	Adj OR (95% CI)	Author (year)
Poor hygiene conditions					
Poor hygiene conditions		H	1 (0.3–3.4), $P = 0.83$		Dinh (2006)
Frequent disinfection of worksite					
Frequency of worksite disinfection: < twice monthly		F	7.5 (1.6–34.2), $P = 0.01$	5.1 (1.1–24.6). $P = 0.04$	Yang (2012)
Cleaning knife after preparing poultry					
Household uses soap to clean		H	0.3 (0.1–1), $P = 0.06$		Mounts (1999)
Ecological study: markets					
Number of LBMs	NS			BRT weight > 5	Fang (2013)
LBM density	NS			1.1 (1–1.1), $P < 0.001$	Fuller (2014)
LBM closure	NS	M		Red. in daily incidence	Yu (2014)
LBM closure	NS	M		Red. in daily incidence	Wu (2014)
Ecological study: poultry density					
Poultry density (1,000/km^2)	NS			RR = 1 (0.8–1.1), $P = 0.59$	Yupiana (2010)
Ecological study: outbreaks in poultry					
The number of poultry outbreaks	Sick			RR = 1.3 (1.1–1.7), $P = 0.02$	Yupiana (2010)

BRT = boosted regression tree; CI = confidence interval; LBM = live bird market; RR = risk relative. “H.” refers to the health status of poultry (H: healthy; NS: not specified; sick: sick or dead poultry); “L.” refers to the location where exposure (NS: not specified, H: households, M: markets, F: farms, P: premises, including markets, farms, and abattoirs); non-adj OR: odds ratios non-adjusted for other exposures, along with 95% confidence interval and P value; adj OR: odds ratios adjusted for other exposures, along with 95% confidence interval and P value; If other measures of association than odds ratios are assessed, they are mentioned in the table.

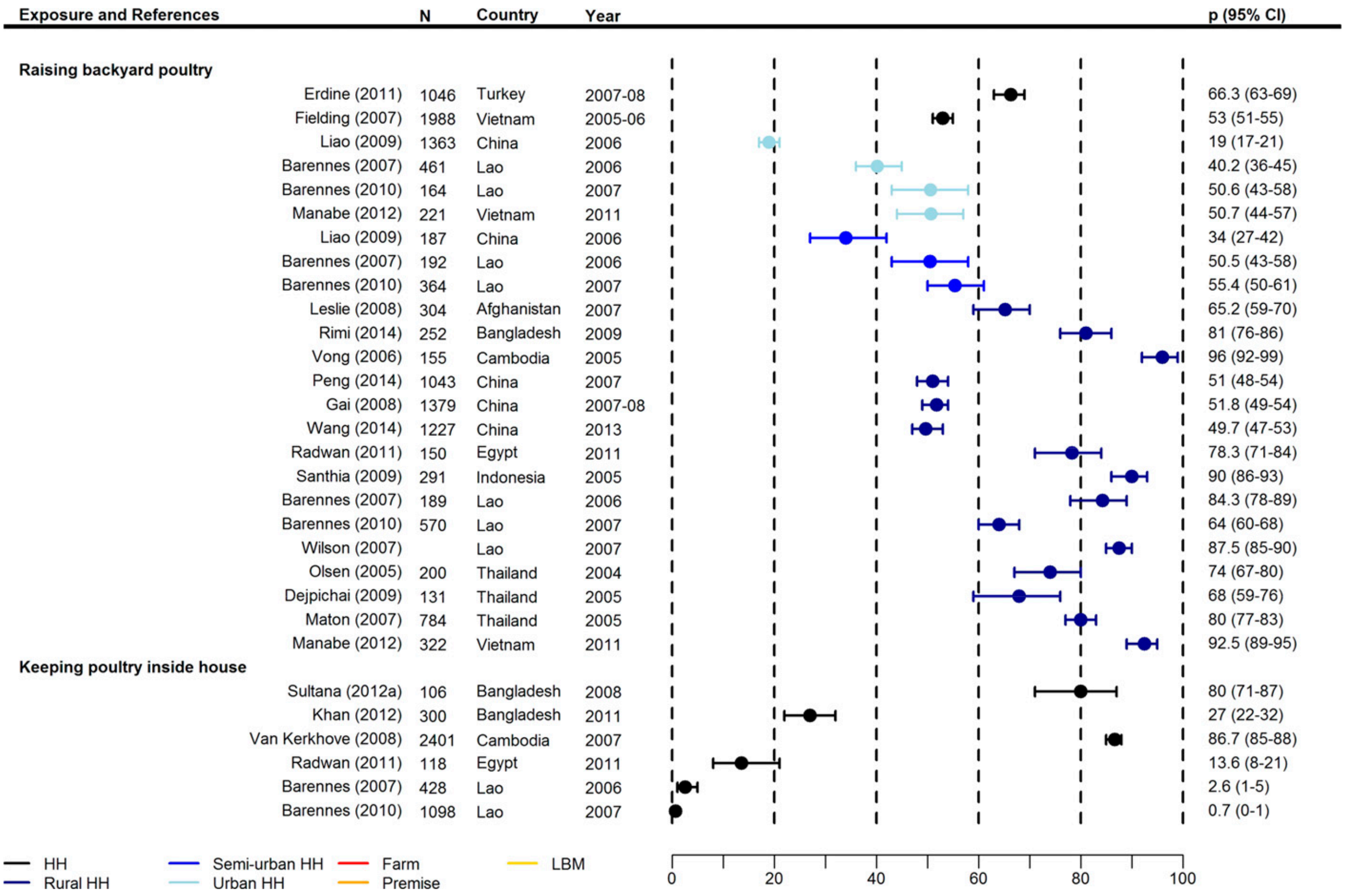

Supplemental Figure 1. Prevalence of practices related to backyard poultry rearing. Premise includes farms, markets, and abattoirs. HH = household.

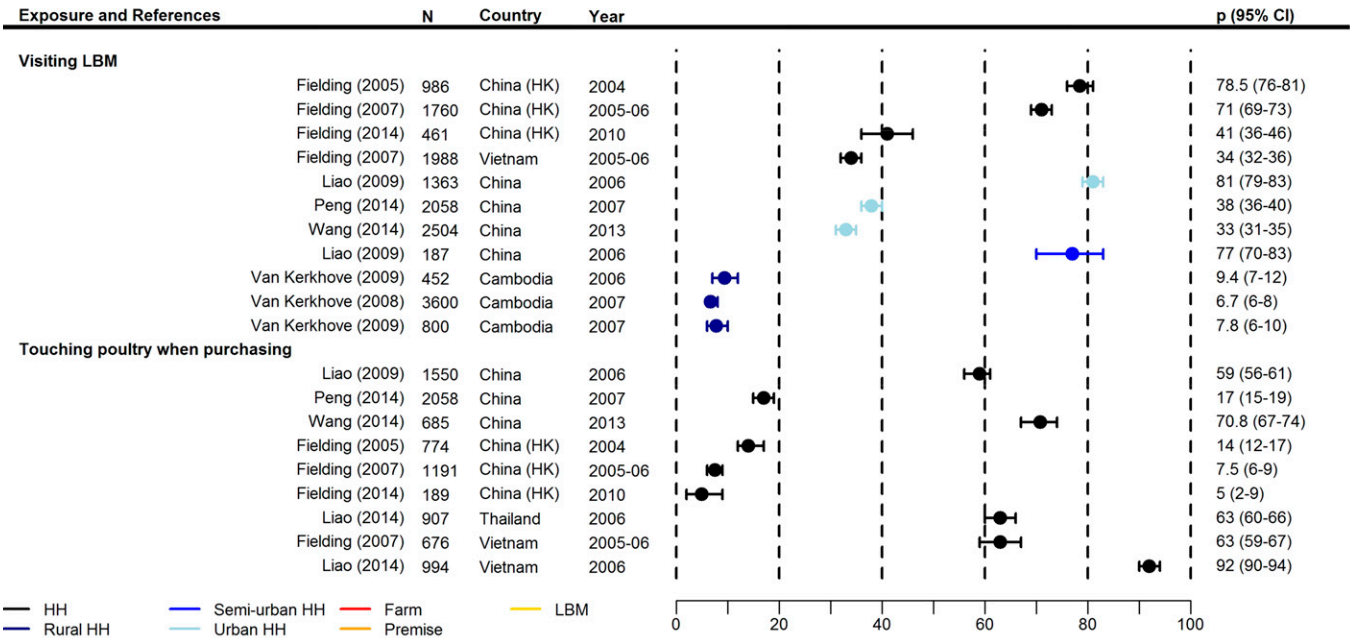

Supplemental Figure 2. Prevalence of practices related to purchase of poultry in live bird markets. Premise includes farms, markets, and abattoirs. HH = household.

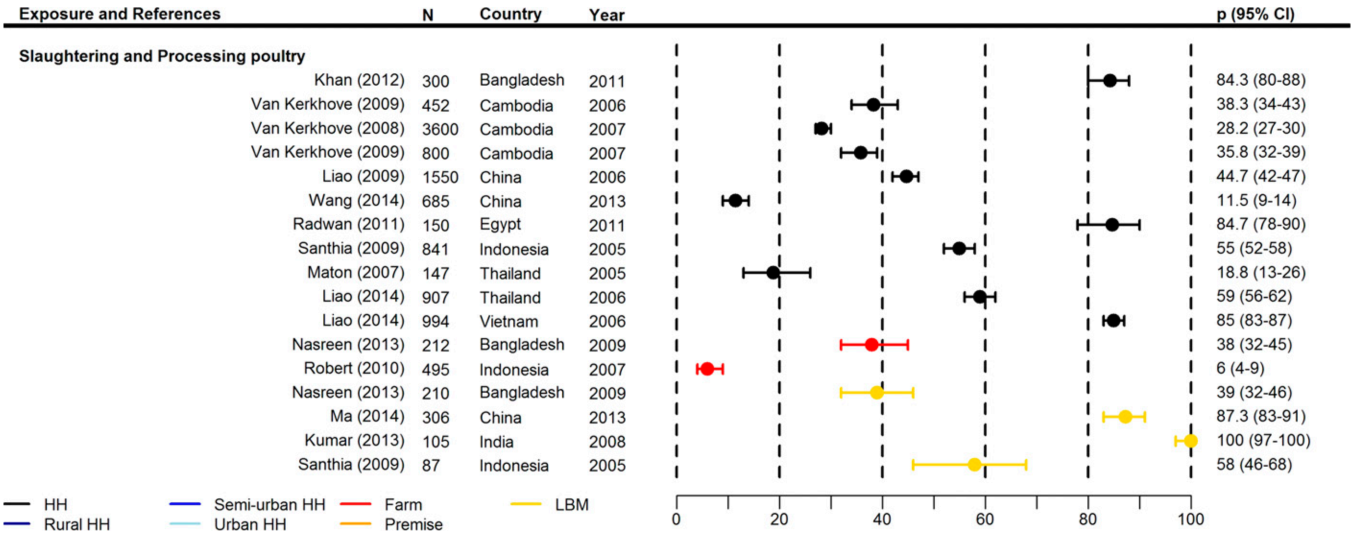

Supplemental Figure 3. Prevalence of practices related to the slaughtering and processing of poultry. Premise includes farms, markets, and abattoirs. HH = household.

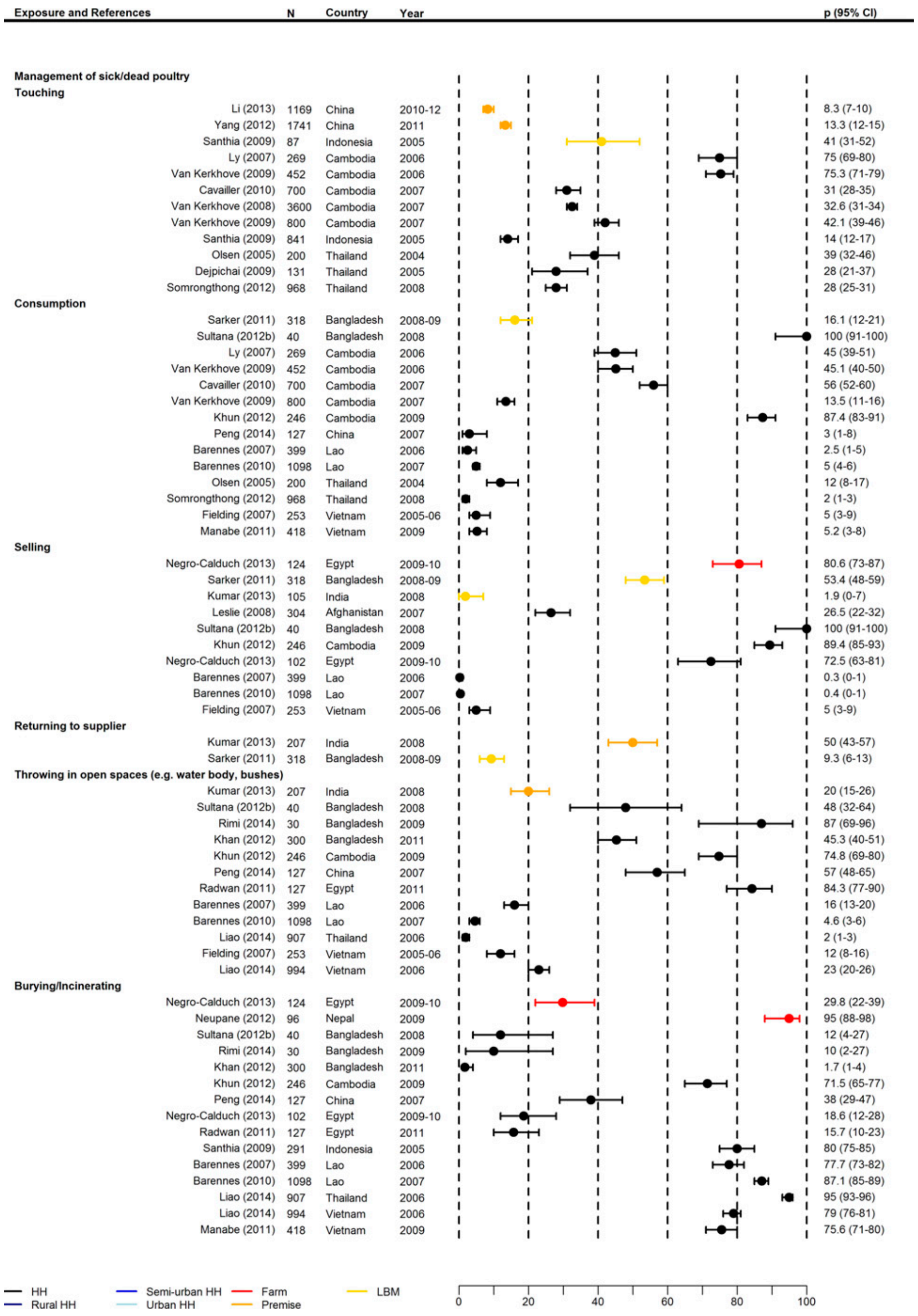

SUPPLEMENTAL FIGURE 4. Prevalence of practices related to the management of sick and dead poultry. Premise includes farms, markets, and abattoirs. HH = household.

Exposure and References	N	Country	Year	p (95% CI)
**Hygiene**				
**Washing hands after contact with poultry**				
Sarker (2011)	318	Bangladesh	2008-09	68.1 (63-73)
Rimi (2014)	30	Bangladesh	2009	4 (0-17)
Radwan (2011)	118	Egypt	2011	98.3 (94-100)
Maton (2007)	147	Thailand	2005	80 (73-86)
Somrongthong (2012)	691	Thailand	2008	99 (98-100)
Manabe (2011)	418	Vietnam	2009	96 (94-98)
Manabe (2012)	322	Vietnam	2011	98.8 (97-100)
**Wearing gloves**				
Nasreen (2013)	212	Bangladesh	2009	27 (21-33)
Robert (2010)	495	Indonesia	2007	10 (8-13)
Kayali (2011)	72	Lebanon	2010	1.4 (0-7)
Neupane (2012)	96	Nepal	2009	30.2 (21-40)
Yu (2013)	305	China	2009-10	54.1 (48-60)
Yang (2012)	1741	China	2011	47 (45-49)
Ma (2014)	306	China	2013	60 (54-66)
Radwan (2011)	127	Egypt	2011	1.6 (0-6)
Kayali (2011)	128	Lebanon	2010	1.6 (0-6)
Somrongthong (2012)	691	Thailand	2008	0.87 (0-2)
**Wearing facemask**				
Nasreen (2013)	212	Bangladesh	2009	66 (59-72)
Robert (2010)	495	Indonesia	2007	14.1 (11-18)
Kayali (2011)	72	Lebanon	2010	5.5 (2-14)
Neupane (2012)	96	Nepal	2009	27.1 (19-37)
Yu (2013)	305	China	2009-10	45.9 (40-52)
Yang (2012)	1741	China	2011	42 (40-44)
Sarker (2011)	318	Bangladesh	2008-09	16.7 (13-21)
Ma (2014)	306	China	2013	20 (16-25)
Sutanto (2013)	100	Indonesia	2012	13 (7-21)
Kuo (2011)	177	Taiwan	2009-10	45.2 (38-53)
Radwan (2011)	127	Egypt	2011	1.6 (0-6)
Kayali (2011)	128	Lebanon	2010	0 (0-3)
Maton (2007)	147	Thailand	2005	2 (0-6)
Somrongthong (2012)	691	Thailand	2008	1.7 (1-3)
**Wearing aprons / Changing clothes**				
Nasreen (2013)	212	Bangladesh	2009	34 (28-41)
Kayali (2011)	72	Lebanon	2010	4.2 (1-12)
Neupane (2012)	96	Nepal	2009	3.1 (1-9)
Yu (2013)	305	China	2009-10	88.9 (85-92)
Ma (2014)	306	China	2013	80 (75-84)
Samaan (2012)	34	Indonesia	2008	15 (5-31)
Samaan (2012)	29	Indonesia	2009	55 (36-74)
Samaan (2011)	37	Indonesia	2010	29.7 (16-47)
Radwan (2011)	118	Egypt	2011	5.1 (2-11)
Kayali (2011)	128	Lebanon	2010	0 (0-3)
**Wearing boots**				
Nasreen (2013)	212	Bangladesh	2009	16 (11-22)
Kayali (2011)	72	Lebanon	2010	15.3 (8-26)
Neupane (2012)	96	Nepal	2009	7.3 (3-14)
Ma (2014)	306	China	2013	80 (75-84)
Samaan (2012)	34	Indonesia	2008	65 (46-80)
Samaan (2012)	29	Indonesia	2009	55 (36-74)
Samaan (2011)	37	Indonesia	2010	29.7 (16-47)
Kayali (2011)	128	Lebanon	2010	6.3 (3-12)
**Rinsing/washing equipment after use**				
Neupane (2012)	96	Nepal	2009	40.6 (31-51)
Samaan (2012)	34	Indonesia	2008	38 (22-56)
Samaan (2012)	29	Indonesia	2009	62 (42-79)
Samaan (2011)	37	Indonesia	2010	18.9 (8-35)
Sutanto (2013)	100	Indonesia	2012	100 (96-100)
Rimi (2014)	30	Bangladesh	2009	33 (17-53)
Radwan (2011)	127	Egypt	2011	99.3 (96-100)

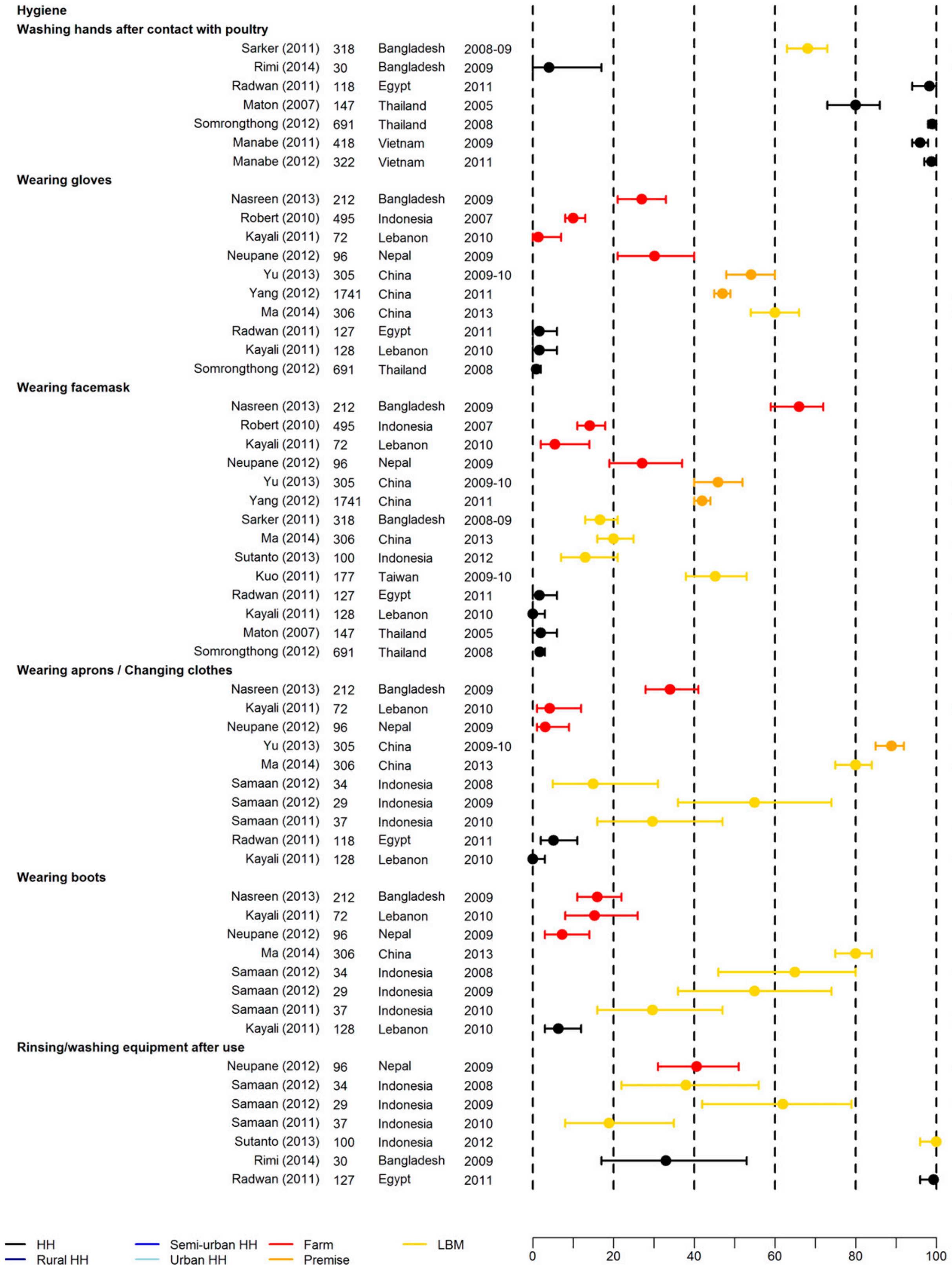

Supplemental Figure 5. Prevalence of preventive practices. Premise includes farms, markets, and abattoirs. HH = household.

REFERENCES

1. Sterne JAC, Higgins JPT, Reeves BC. *A Cochrane Risk Of Bias Assessment Tool: for Non-Randomized Studies of Interventions (ACROBAT-NRSI), Version 1.0.0*; 2014.
2. Zhou L, Liao Q, Dong L, et al. Risk factors for human illness with avian influenza A (H5N1) virus infection in China. *Journal of Infectious Diseases* 2009; *199:* 1726–34.
3. Dinh PN, Long HT, Tien NT, et al. Risk factors for human infection with avian influenza A H5N1, Vietnam, 2004. *Emerg Infect Dis 2006; 12:* 1841–7.
4. Li J, Chen J, Yang G, et al. Case-control study of risk factors for human infection with avian influenza A(H7N9) virus in Shanghai, *China*, 2013. *Epidemiology and Infection 2014:* 1–7.
5. Liu B, Havers F, Chen EF, et al. Risk Factors for Influenza A(H7N9) Disease-China, 2013. *Clinical infectious diseases : an official publication of the Infectious Diseases Society of America 2014; 59:* 787–94.
6. He F, Zhang M, Wang XY, et al. Distinct Risk Profiles for Human Infections with the Influenza A(H7N9) Virus among Rural and Urban Residents: Zhejiang Province, China, 2013. *PLoS One 2014;* 9.
7. Ai J, Huang Y, Xu K, et al. Case-control study of risk factors for human infection with influenza A(H7N9) virus in Jiangsu Province, China, 2013. *Euro surveillance : bulletin Europeen sur les maladies transmissibles = European communicable disease bulletin* 2013; *18:* 20510.
8. Mounts AW, Kwong H, Izurieta HS, et al. Case-control study of risk factors for avian influenza A (H5N1) disease, Hong Kong, 1997. *J Infect Dis* 1999; *180:* 505–8.
9. Bridges CB, Lim W, Hu-Primmer J, et al. Risk of influenza A (H5N1) infection among poultry workers, Hong Kong, 1997-1998. *J Infect Dis* 2002; *185:* 1005–10.
10. Vong S, Ly S, Van Kerkhove MD, et al. Risk factors associated with subclinical human infection with avian influenza A (H5N1) virus–Cambodia, 2006. *J Infect Dis* 2009; *199:* 1744–52.
11. Areechokchai D, Jiraphongsa C, Laosiritaworn Y, Hanshaoworakul W, O'Reilly M, Centers for Disease Control and Prevention. Investigation of avian influenza (H5N1) outbreak in humans–Thailand, 2004. *MMWR Morbidity and mortality weekly report* 2006; *55:* 3–6.
12. Huo X, Zu RQ, Qi X, et al. Seroprevalence of avian influenza A (H5N1) virus among poultry workers in Jiangsu Province, China: an observational study. *BMC infectious diseases* 2012; 12.
13. Yu Q, Liu L, Pu J, et al. Risk perceptions for avian influenza virus infection among poultry workers, China. *Emerging infectious diseases* 2013; *19:* 313–6.
14. Yu H, Wu JT, Cowling BJ, et al. Effect of closure of live poultry markets on poultry- to-person transmission of avian influenza A H7N9 virus: an ecological study. *Lancet* 2014; *383:* 541–8.
15. Wu P, Jiang H, Wu JT, et al. Poultry Market Closures and Human Infection with Influenza A(H7N9) Virus, China, 2013-14. *Emerging infectious diseases* 2014; *20:* 1891–4.
16. Gomaa MR, Kayed AS, Elabd MA, et al. Avian Influenza A(H5N1) and A(H9N2) Seroprevalence and Risk Factors for Infection Among Egyptians: A Prospective, Controlled Seroepidemiological Study. *Journal of Infectious Diseases* 2014.
17. Wang X, Fang S, Lu X, et al. Seroprevalence to avian influenza A (H7N9) virus among poultry workers and the general population in southern China: a longitudinal study. *Clinical infectious diseases : an official publication of the Infectious Diseases Society of America* 2014; *59:* e76–83.
18. Yang P, Ma C, Shi W, et al. A serological survey of antibodies to H5, H7 and H9 avian influenza viruses amongst the duck-related workers in Beijing, China. *PLoS One* 2012; *7:* e50770.
19. Ahad A, Thornton RN, Rabbani M, et al. Risk factors for H7 and H9 infection in commercial poultry farm workers in provinces within Pakistan. *Prev Vet Med* 2014; *117:* 610–4.
20. Dinh PN, Long HT, Tien NT, et al. Risk factors for human infection with avian influenza A H5N1, Vietnam, 2004. *Emerging infectious diseases 2006; 12:* 1841–7.
21. Fournie G, Pfeiffer DU. Can closure of live poultry markets halt the spread of H7N9? *Lancet* 2014; *383:* 496–7.
22. Yupiana Y, de Vlas SJ, Adnan NM, Richardus JH. Risk factors of poultry outbreaks and human cases of H5N1 avian influenza virus infection in West Java Province, Indonesia. *Int J Infect Dis* 2010; *14:* e800–5.
23. Cavailler P, Chu S, Ly S, et al. Seroprevalence of anti-H5 antibody in rural Cambodia, 2007. *J Clin Virol 2010; 48:* 123–6.
24. Li LH, Yu Z, Chen WS, et al. Evidence for H5 avian influenza infection in Zhejiang province, China, 2010-2012: a cross-sectional study. *J Thorac Dis* 2013; *5:* 790–6.
25. Fang LQ, Li XL, Liu K, et al. Mapping spread and risk of avian influenza A (H7N9) in China. *Scientific reports* 2013; *3:* 2722.
26. Fuller T, Havers F, Xu C, et al. Identifying areas with a high risk of human infection with the avian influenza A (H7N9) virus in East Asia. *J Infection* 2014; *69:* 174–81.
27. Leslie T, Billaud J, Mofleh J, Mustafa L, Yingst S. Knowledge, attitudes, and practices regarding avian influenza (H5N1), Afghanistan. *Emerging infectious diseases* 2008; *14:* 1459–61.
28. Vong S, Coghlan B, Mardy S, et al. Low frequency of poultry-to-human H5NI virus transmission, southern Cambodia, 2005. *Emerg Infect Dis 2006; 12:* 1542–7.
29. Liao Q, Lam WT, Leung GM, Jiang C, Fielding R. Live poultry exposure, Guangzhou, China, 2006. *Epidemics 2009; 1:* 207–12.
30. Wang LP, Cowling BJ, Wu P, et al. Human Exposure to Live Poultry and Psychological and Behavioral Responses to Influenza A(H7N9), China. *Emerging infectious diseases* 2014; *20:* 1296–305.
31. Radwan GN, Wahid WY, El-Derwy D, El-Rabat M. Knowledge, attitudes, and practices of avian influenza among backyard poultry breeders in Fayoum Governorate, Egypt. *The Journal of the Egyptian Public Health Association* 2011; *86:* 104–10.
32. Santhia K, Ramy A, Jayaningsih P, et al. Avian influenza A H5N1 infections in Bali Province, Indonesia: a behavioral, virological and seroepidemiological study. *Influenza Other Respi Viruses* 2009; *3:* 81–9.
33. Barennes H, Harimanana AN, Lorvongseng S, Ongkhammy S, Chu C. Paradoxical risk perception and behaviours related to Avian Flu outbreak and education campaign, Laos. *BMC infectious diseases* 2010; *10:* 294.
34. Wilson RT. Numbers, ownership, production and diseases of poultry in the Lao People's Democratic Republic. *World Poultry Sci J* 2007; *63:* 655–63.
35. Maton T, Butraporn P, Kaewkangwal J, Fungladda W. Avian influenza protection knowledge, awareness, and behaviors in a high-risk population in Suphan Buri Province, Thailand. *The Southeast Asian journal of tropical medicine and public health* 2007; *38:* 560–8.
36. Somrongthong R, Beaudoin A, Bender J, et al. Use of personal protective measures by Thai households in areas with avian influenza outbreaks. *Zoonoses and Public Health* 2012; *59:* 339–46.
37. Edirne T, Avci DK, Dagkara B, Aslan M. Knowledge and anticipated attitudes of the community about bird flu outbreak in Turkey, 2007-2008: a survey-based descriptive study. *Int J Public Health* 2011; *56:* 163–8.
38. Rimi NA, Sultana R, Ishtiak-Ahmed K, et al. Poultry Slaughtering Practices in Rural Communities of Bangladesh and Risk of Avian Influenza Transmission: A Qualitative Study. *EcoHealth* 2014; *11:* 83–93.
39. Peng Z, Wu P, Ge L, et al. Rural villagers and urban residents exposure to poultry in China. *PLoS One* 2014; *9:* e95430.
40. Gai R, Wang X, Zhang Y, Xu L. Knowledge and practice of poultry handling and living environments of rural residents in China. *Bioscience trends* 2008; *2:* 61–3.
41. Fielding R, Lam WW, Ho EY, Lam TH, Hedley AJ, Leung GM. Avian influenza risk perception, Hong Kong. *Emerging infectious diseases* 2005; *11:* 677–82.
42. Fielding R, Bich TH, Quang LN, et al. Live poultry exposures, Hong Kong and Hanoi, 2006. *Emerging infectious diseases 2007; 13:* 1065–7.
43. Fielding R, Cowling BJ, Liao Q, Lam WW. Behavioural changes in relation to risk perception and prevention of avian and human influenza in Hong Kong, 2006 to 2010. *Hong Kong medical journal = Xianggang yi xue za zhi / Hong Kong Academy of Medicine* 2014; 20 Suppl *4:* 26–8.
44. Barennes H, Martinez-Aussel B, Vongphrachanh P, Strobe M. Avian influenza risk perceptions, *Laos. Emerging infectious diseases* 2007; *13:* 1126–8.

45. Olsen SJ, Laosiritaworn Y, Pattanasin S, Prapasiri P, Dowell SF. Poultry-handling practices during avian influenza outbreak, Thailand. *Emerging infectious diseases* 2005; *11:* 1601–3.
46. Dejpichai R, Laosiritaworn Y, Phuthavathana P, et al. Seroprevalence of antibodies to avian influenza virus A (H5N1) among residents of villages with human cases, Thailand, 2005. *Emerging infectious diseases* 2009; 15: 756–60.
47. Liao QY, Lam WWT, Bich TH, Dang VT, Fielding R. Comparison of behaviors regarding live poultry exposure among rural residents in Vietnam and Thailand. *J Infect Dev Ctries* 2014; *8:* 526–34.
48. Manabe T, Pham TP, Vu VC, et al. Impact of educational intervention concerning awareness and behaviors relating to avian influenza (H5N1) in a high-risk population in Vietnam. *PLoS One* 2011; *6:* e23711.
49. Manabe T, Tran TH, Doan ML, et al. Knowledge, attitudes, practices and emotional reactions among residents of avian influenza (H5N1) hit communities in Vietnam. *PLoS One* 2012; *7:* e47560.
50. Sultana R, Nahar N, Rimi NA, et al. Backyard poultry raising in Bangladesh: a valued resource for the villagers and a setting for zoonotic transmission of avian influenza. A qualitative study. *Rural and remote health* 2012; *12:* 1927.
51. Sultana R, Rimi NA, Azad S, et al. Bangladeshi backyard poultry raisers' perceptions and practices related to zoonotic transmission of avian influenza. *J Infect Dev Ctries* 2012; *6:* 156–65.
52. Khan MSI, Akbar SMF, Hossain ST, Mahatab M, Hossain MM, Idrus Z. Possible Route of Transmission of Highly Pathogenic Avian Influenza Virus Type H5N1 in Family Poultry at Rural Bangladesh. *Pak Vet J* 2012; *32:* 112–6.
53. Ly S, Van Kerkhove MD, Holl D, Froehlich Y, Vong S. Interaction between humans and poultry, rural Cambodia. *Emerging infectious diseases* 2007; *13:* 130–2.
54. Van Kerkhove MD, Ly S, Guitian J, et al. Changes in poultry handling behavior and poultry mortality reporting among rural Cambodians in areas affected by HPAI/H5N1. *PLoS One* 2009; *4:* e6466.
55. Van Kerkhove MD, Ly S, Holl D, et al. Frequency and patterns of contact with domestic poultry and potential risk of H5N1 transmission to humans living in rural Cambodia. *Influenza Other Respi Viruses* 2008; *2:* 155–63.
56. Khun M, Heng C, Md H-O-R, Kasuya H, Sakamoto J. Knowledge, attitudes and practices towards avian influenza A (H5N1) among Cambodian women: a cross-sectional study. *Asian Pac J Trop Med* 2012; *5:* 727–34.
57. Negro-Calduch E, Elfadaly S, Tibbo M, Ankers P, Bailey E. Assessment of biosecurity practices of small-scale broiler producers in central Egypt. *Preventive Veterinary Medicine* 2013; *110:* 253–62.
58. Kayali G, Barbour E, Dbaibo G, et al. Evidence of infection with H4 and H11 avian influenza viruses among Lebanese chicken growers. *PLoS One* 2011; *6:* e26818.
59. Nasreen S, Khan SU, Azziz-Baumgartner E, et al. Seroprevalence of Antibodies against Highly Pathogenic Avian Influenza A (H5N1) Virus among Poultry Workers in Bangladesh, 2009. *PLoS One 2013;* 8.
60. Robert-Dy Ry van Beest Holle M, Setiawaty V, Pangesti KNA, Sedyaningsih ER. Seroprevalence of Avian Influenza a/H5n1 among Poultry Farmers in Rural Indonesia, 2007. *The Southeast Asian journal of tropical medicine and public health* 2010; *41:* 1095–103.
61. Neupane D, Khanal V, Ghimire K, Aro AR, Leppin A. Knowledge, attitudes and practices related to avian influenza among poultry workers in Nepal: a cross sectional study. *BMC infectious diseases* 2012; 12.
62. Sarker S, Talukder S, Chowdhury EH, Das PM. Knowledge, attitudes and practices on biosecurity of workers in live bird markets at Mymensingh, Bangladesh. *Journal of Agricultural and Biological Science* 2011; *6:* 12–7.
63. Ma X, Liao Q, Yuan J, et al. Knowledge, attitudes and practices relating to influenza A(H7N9) risk among live poultry traders in Guangzhou City, China. *BMC infectious diseases* 2014; *14:* 554.
64. Kumar SC, Ramesh N, Sreevatsan S, et al. Knowledge, attitudes, and poultry-handling practices of poultry workers in relation to avian influenza in India. *Indian journal of occupational and environmental medicine* 2013; *17:* 16–21.
65. Samaan G, Hendrawati F, Taylor T, et al. Application of a healthy food markets guide to two Indonesian markets to reduce transmission of "avian flu". *Bulletin of the World Health Organization* 2012; *90:* 295–300.
66. Samaan G, Gultom A, Indriani R, Lokuge K, Kelly PM. Critical control points for avian influenza A H5N1 in live bird markets in low resource settings. *Preventive Veterinary Medicine* 2011; *100:* 71–8.
67. Sutanto YC. *Highly Pathogenic Avian Influenza Knowledge, Attitudes, and Practices Study among Live Bird Market Workers in Jakarta – Indonesia* [Master's Thesis]. Fort Collins, Colorado: Colorado State University; 2013.
68. Kuo PC, Huang JH, Liu MD. Avian influenza risk perception and preventive behavior among traditional market workers and shoppers in Taiwan: practical implications for prevention. *PLoS One* 2011; *6:* e24157.
